# Environment ensemble models for genomic prediction in common bean (*Phaseolus vulgaris* L.)

**DOI:** 10.1002/tpg2.70057

**Published:** 2025-06-12

**Authors:** Isabella Chiaravallotti, Owen Pauptit, Valerio Hoyos‐Villegas

**Affiliations:** ^1^ Department of Plant, Soil and Microbial Sciences Michigan State University East Lansing Michigan USA; ^2^ Department of Plant Science McGill University Montreal Quebec Canada; ^3^ School of Informatics University of Edinburgh Edinburgh Scotland UK

## Abstract

For important food crops such as the common bean (*Phaseolus vulgaris*, L.), global demand continues to outpace the rate of genetic gain for quantitative traits. In this study, we leveraged the multi‐environment trial (MET) dataset from the cooperative dry bean nursery (CDBN) to investigate the use of ensemble models for genomic prediction. This set spans 70 locations and 30 years, and accounts for over 150 phenotypes and hundreds of genotypes sequenced for 1.2 million single nucleotide polymorphism markers. We tested three models (linear regression, ridge regression, and neural networks). Each of the three models was implemented using three different approaches: (1) combining all data into one model (singular model), (2) all available single locations were used to train individual submodels comprising one ensemble model (ensemble model), and (3) optimized sets of single locations were used to train individual submodels comprising one ensemble model (optimized ensemble model). The optimized ensemble approach worked best for low‐variance locations because the model variance was reduced by averaging across submodels in the ensemble. For models with low prediction accuracy, the ensemble approach can increase accuracy. In certain locations, prediction accuracy was able to overcome narrow‐sense heritability, indicating that genomic selection is more efficient than phenotypic selection in these locations. This study indicates that breeding program collaboration can be a way to bypass the bottleneck of low data volume, as pooled data from the CDBN MET produced prediction accuracies of 0.70 for days to flowering, 0.54 for days to maturity, 0.95 for seed weight, and 0.67 for seed yield in individual locations.

## Core Ideas

- Genomic prediction ensemble models with low variance multi‐environment data result in increased prediction accuracy.
- Genomic prediction ensemble models can be transferred between environments with robust multi‐location training sets.
- Correlation among environments should be considered to achieve a useful level of prediction accuracy.
- Ensembles can be used to increase the accuracy of neural networks for genomic prediction.
- Genomic prediction ensembles achieve higher selection accuracy in locations with very low narrow‐sense heritability.

**Abbreviations**
CDBN: Cooperative Dry Bean Nursery
DF: days to flowering
DM: days to maturity
ELR: ensemble linear regression
ENN: ensemble neural network
ERR: ensemble ridge regression
GGE: genotype plus genotype‐by‐environment
GP: genomic prediction
GS: genomic selection
LR: linear regression
MET: multi‐environment trial
NN: neural network
OLR: optimized ensemble linear regression
ONN: optimized ensemble neural network
ORR: optimized ensemble ridge regression
PCA: principal components analysis
QTL: quantitative trait locus
RFLP: restriction fragment length polymorphism
RR: ridge regression
SLR: singular linear regression
SNN: singular neural network
SNP: single nucleotide polymorphism
SRR: singular ridge regression
SW: seed weight
SY: seed yield

## 1 INTRODUCTION

### 1.1 Genomic prediction

Genomic prediction (GP) can be likened to Plato's *Allegory of the Cave*. In the allegory, a person in a cave observes shadows cast on the cave's walls by objects outside of the cave. The shadows represent what we perceive, and the activities outside the cave are the world's objective truth. Applied to genetics, we can consider the phenotype to be the shadow cast by the genotype, to the extent that the phenotype is not largely explained by the genotype (i.e., low linkage). The expressed phenotype is liable to shift and change due to its plastic nature (i.e., low heritability), just as the shadows in Plato's cave shift depending on the position of the sun outside of the cave. In the context of plant breeding, the person observing the shadows on the wall is the breeder. Breeders are tasked with the difficult and intensive challenge of interpreting phenotypes across different environments and identifying the best genotypes. Thus, the optimal tool for a breeder is one that minimizes the noise caused by the environment and increases the signal that constitutes the genotype.

GP was first implemented in 1994 by Professor Rex Bernardo at the University of Minnesota (Bernardo, 1994) using restriction fragment length polymorphism (RFLP) data from parents to predict the performance of single crosses in maize. Bernardo found that it is, in fact, possible to use RFLPs to predict the performance of crosses. Further, the accuracy of the prediction model was slightly higher when dominance effects and coefficients of co‐ancestry were included in the model, foreshadowing the plethora of subsequent studies aimed at discovering the ideal method for modeling individual marker effects. Similarly, Meuwissen et al. (2001) estimated the effects of a dense marker panel to predict the breeding value of simulated individuals. This was revolutionary because prior to dense marker array information, breeding values could only be estimated using phenotypic records and progeny tests, which were time and resource intensive. In many crop plants, selection is carried out in the parents per se, so progeny tests are not always necessary to estimate breeding values. However, generation of breeding values was particularly useful in animal breeding, and genetic gain for milk yield in dairy cattle has greatly benefited from the use of GP (Doublet et al., 2019; Garcia‐Ruiz et al., 2016).

It should be noted that there are few instances where GP has been demonstrated empirically in food crops. Theory and simulation (Hoyos‐Villegas et al., 2019) show that by implementing genomic selection (GS), breeders may shorten the breeding cycle while achieving higher selection accuracy compared to traditional methods. To highlight a few examples, Bandillo et al. (2023) conducted a selection experiment using GP in the field and compared it to phenotypic selection with random selection as a control. They found that lines selection based on genomic estimated breeding value (GEBV) had a higher seed yield (SY) than those selected phenotypically, but GEBV had a more dramatic depletion of genetic variance than phenotypic selection. Das et al. (2020) conducted rapid cycle GS on maize and compared GEBV selections to check hybrids. They found that after three cycles of rapid‐cycle GS, grain yield was higher than that of check hybrids. However, unlike Bandillo et al. (2023), they did not find a notable negative impact on genetic variance. Massman et al. (2013) compared GS to marker‐assisted selection in maize. They found that using all available markers (in other words, implementing GS) resulted in higher yield and stover index gains than using only a small set of markers with significant effects (in other words, using marker‐assisted selection).

Alemu et al. (2024) give a comprehensive overview of the progress in GS during the past 20 years, reviewing machine learning model optimization studies, training set optimization studies, marker density, phenotyping, and empirical validation conducted since the early Bernardo (1994) and Meuwissen et al. (2001) studies. A common thread through many of these studies is the use of machine learning models to make use of data across geographic locations and across time. This includes modeling genotype‐by‐environment interaction (Heslot et al., 2014; Mageto et al., 2020; Semagn et al., 2022), increasing selection intensity via increased population size by using sparse testing to predict the value of untested genotypes (Atanda et al., 2021; Crespo‐Herrera et al., 2021; Isidro y Sanchez & Akdemir, 2021; Persa et al., 2023; Montesinos‐Lopez et al., 2023), leveraging historical data to build a more comprehensive dataset (Hao et al., 2019; Sarinelli et al., 2019), identifying major quantitative trait locus (QTL) that are stable across environments (Jannink et al., 2010), and understanding at what stage of breeding to implement GS (Biswas et al., 2023; Chiaravallotti et al., 2024; Kadam et al., 2016; Rembe et al., 2022). Because model coefficients are computed according to the dataset used to train the model, a key step for breeders wishing to implement GS is the construction of the training set. Isidro y Sanchez and Akdemir (2021) points out that although training set optimization is an active area of research, more study is needed to determine whether certain claims about training set size, trait heritability, and genetic relatedness hold true across multiple breeding programs. Further, training set optimization for multi‐environment trials (METs) and sparse testing is emerging as a key area for future investigation (Atanda et al., 2021; Jarquin et al., 2020; Montesinos‐Lopez et al., 2023).

### 1.2 Genomic prediction in common bean

A handful of studies have assessed the potential of GS for common bean (*Phaseolus vulgaris* L.) breeding. Lin et al. (2023) conducted a simulation study to investigate GS in common bean and concluded that depending on the genetic architecture of the trait at hand, GS may be able to achieve a higher rate of genetic gain than traditional phenotype‐based methods. Chiaravallotti et al. (2024) also highlighted the significance of trait architecture on the success of GS, and they concluded that certain breeding strategies (e.g., single seed descent or the pedigree method) are more amenable to GS than others. Further, Chiaravallotti et al. (2024) conducted a simulation study exploring GS in common bean and indicate that the genetic makeup of the training set in terms of allele frequencies is a key factor when training a GP model for GS. In 2018, Barili et al. tested several models for predicting grain yield, plant architecture, and grain aspect (Barili et al., 2018). They found that ridge regression (RR) was the best model for polygenic traits such as SY. They used only 377 single nucleotide polymorphism (SNP) markers, but reached moderate accuracy of 0.25, 0.28, and 0.47 for plant architecture, grain aspect, and grain yield, respectively. Keller et al. (2020) used historical data to predict four traits using data from twelve different trials and 5820 SNP markers. Across all traits, the prediction accuracy ranged from 0.3 to 0.8. By accumulating trial data over time, they leveraged historical data to better model genotype‐by‐environment (G × E) and to increase prediction accuracy. They concluded that GP is a powerful tool for bean breeding; however, acquiring adequate phenotypic data will be a bottleneck for GP implementation. Therefore, breeders’ ability to phenotype a subset of genotypes and predict the value of remaining genotypes in a given environment (without phenotyping) will be an important approach moving forward. Similarly, Shao et al. (2022) investigated the utility of diverse landrace accessions as a training set for predicting the value of breeding lines. They found that including breeding lines in the training set increased prediction accuracy for traits such as 100‐seed weight (SW), SY, and days to flowering (DF). The increase in accuracy observed when including breeding lines in the training set, in addition to landrace accessions, indicates the importance of relatedness between the training set and the test set. They also concluded that while breeders can use diverse germplasm accessions for training, it is best to include training data from inside the breeding program. Therefore, bean breeders need to be strategic about which lines they genotype and phenotype in their program to construct the ideal training set. This study aims to assess the utility of accumulated data across locations for training set construction.

**Core Ideas**

- Genomic prediction ensemble models with low variance multi‐environment data result in increased prediction accuracy.
- Genomic prediction ensemble models can be transferred between environments with robust multi‐location training sets.
- Correlation among environments should be considered to achieve a useful level of prediction accuracy.
- Ensembles can be used to increase the accuracy of neural networks for genomic prediction.
- Genomic prediction ensembles achieve higher selection accuracy in locations with very low narrow‐sense heritability.

### 1.3 Genomic prediction as a tool in multi‐environment trials

GP can greatly reduce efforts and resources required to conduct METs. METs are an essential part of plant breeding because they allow breeders to identify which genotypes are (1) stable across multiple environments or (2) uniquely adapted to a specific environment. Testing every genotype in every environment requires space, time, resources, and labor. However, GP can be used as a tool to make METs manageable by using sparse testing, a method that allows breeders to test only some genotypes in each environment while simply predicting the value of the remaining untested genotypes (Jarquin et al., 2020). Jarquin et al. (2020) also points out that a sparse testing method allows breeders to expand the number of genotypes they evaluate without increasing their costs, allowing them to increase the intensity of selection in their program effectively. In order to implement sparse testing with GP, breeders must be able to transfer models across environments with reliable accuracy.

The goals of this study were to (1) identify how model accuracy varies across common bean growing locations, (2) assess the utility of ensemble models for estimating the value of previously observed genotypes in an unobserved environment, and (3) determine the relationship between different common bean growing environments causing variation in model accuracies.

## 2 MATERIALS AND METHODS

### 2.1 Dataset

The dataset used for this study comes from the Cooperative Dry Bean Nursery (CDBN) MET (MacQueen et al., 2020). The MET includes 500 entries grown across 70 locations over 34 years. Together, those participating in the CDBN collected data for over 150 traits. The dataset is relatively sparse, with a given entry being grown, on average, at 19 locations for 2 years.

The traits of interest in this study were SY, SW, days to maturity (DM), and DF. Locations included in the dataset were California (CA), Idaho (ID), Michigan (MI), Montana (MT), North Dakota (ND), Nebraska (NE), Ontario (ON), Washington (WA), Wyoming (WY), New York (NY), Colorado (CO), Arizona (AZ), Alberta (AB), Manitoba (MB), New Mexico (NM), Saskatchewan (SK), Texas (TX), and Puerto Rico (PR). According to MacQueen et al. (2020), phenotype records from 1981 to 2015 were collected, and handwritten records were digitized. Scales for each trait were standardized to make them consistent across years and locations. Due to the sparsity of the dataset, not every location is represented for every trait in this study.

As described in MacQueen et al. (2020), raw sequence data were obtained from the previously sequenced Andean, Durango, and Mesoamerican diversity panels. For previously unsequenced entries, an identical sequencing protocol was followed. DNA was acquired from either young trifoliate leaves, or from seed embryo, if the seed would not germinate. The germplasm was then genotyped using genotyping‐by‐sequencing (Schroeder et al., 2016). Those SNPs with a minor allele frequency > 5% were retained, and any reads with a quality score below 20 were removed, leaving a set of 1,221,540 SNPs. SNPs were imputed using FILLIN in TASSEL (Bradbury et al., 2007).

### 2.2 Data preprocessing

The original dataset contained 1.2 million SNPs, so we performed feature selection to make the modeling computationally feasible. While typically used for dimensionality reduction, principal components analysis (PCA) can also be used for feature selection (Lu et al., 2007; Song et al., 2010). This method involves conducting a PCA and identifying the eigenvectors with the largest eigenvalues, or those that explained the greatest amount of variance in the feature set. The corresponding features (SNPs) are then selected for model training. For each of the four traits used in this study, we conducted feature selection independently, as different features (markers) are assumed to have a variable effect on each trait. From the original 1.2 million SNPs, we were left with 1134 SNPs for DM, 2841 SNPs for DF, 1138 SNPs for SY, and 1134 SNPs for SW. The number of SNPs used for each trait varies because the number of components is limited by the number of observations (rows) in the full dataset. Genotypes were coded in 0‐1‐2 coding, where 0 indicates no copies of the major allele, 1 indicates one copy of the major allele, and 2 indicates two copies of the major allele. After preprocessing, the data were structured as follows: The DF dataset included nine locations, 155 unique genotypes, 2841 SNP markers, and a total of 926 observations across locations. The DM dataset had 18 locations, 325 unique genotypes, 1134 SNP markers, and 3526 total observations across locations. The SW dataset had 18 locations, 325 unique genotypes, 1134 SNP markers, and a total of 3928 total observations. The SY dataset had 19 locations, 327 unique genotypes, 1138 SNP markers, and 4691 total observations across locations.

### 2.3 Cross‐validation

In the literature, four general methods are employed for cross‐validation across different environments (Crossa et al., 2017). First, new unobserved lines can be predicted for previously observed environments. Second, lines observed in some environments can be predicted in previously tested (but different) environments, also called sparse testing. Third, observed genotypes in one or more environments are predicted in untested environments. Fourth, predicting unobserved genotypes in untested environments. This study focuses on the third method, in which we predict the value of previously observed genotypes in an unobserved environment.

**FIGURE 1:**
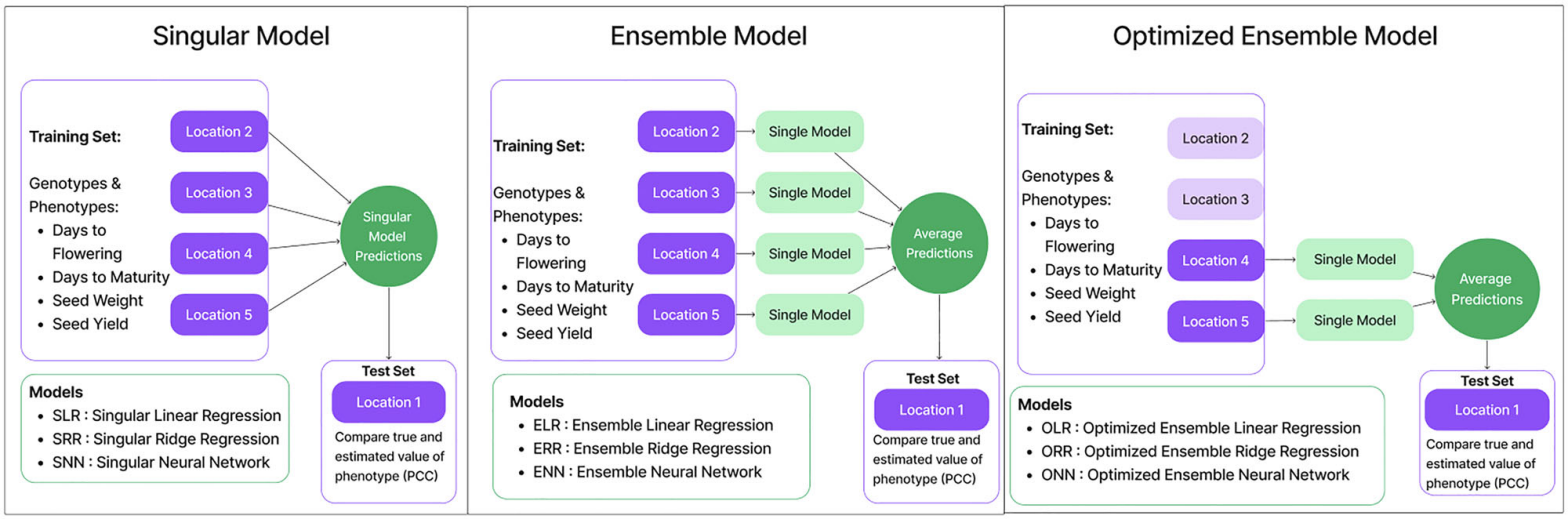
A visual of the three approaches (singular model, ensemble model, and optimized ensemble model) used to train each of the three models (linear regression, ridge regression, and neural network). In total, nine models were tested: singular linear regression (SLR), singular ridge regression (SRR), singular neural network (SNN), ensemble linear regression (ELR), ensemble ridge regression (ERR), ensemble neural network (ENN), optimized ensemble linear regression (OLR), optimized ensemble ridge regression (ORR), and optimized ensemble neural network (ONN). The singular model combines all available data to create a prediction model. The ensemble approach creates an ensemble model using data from all available locations. Each location is used as a training set for the submodels comprising the ensemble. The number of submodels in the ensemble is equal to the number of training locations. The predictions from each submodel in the ensemble are averaged to produce the final output. The optimized ensemble approach creates an ideal ensemble of submodels using data from locations that increase prediction accuracy on the holdout set. The number of submodels in the optimized ensemble was defined by the optimization algorithm and varied by test location. The predictions from each submodel in the optimized ensemble are averaged to produce the final output. PCC, Pearson's correlation coefficient.

We implemented a leave‐one‐out cross‐validation algorithm to test model performance on genotypes in every location present in our dataset. This algorithm leaves out one location to be used as the test set; the remainder of the set is used for model training, accuracy is assessed on the test location, then the first location is replaced, and the next location is left out until all locations have been tested. Three different model training approaches were taken (Figure 1). In this study, model accuracy is computed as the correlation between the true and estimated value of the phenotype for a given observation.

1. First, we trained a singular model in which data from all training locations were combined to train a single model. The singular models will henceforth be referred to as singular linear regression (SLR), singular ridge regression (SRR), and singular neural network (SNN).
2. Second, we trained an ensemble model in which each training location was used to train one submodel, which together comprise an ensemble model. In the ensemble approach, each submodel produces a prediction, and the final prediction for each test observation is the average of all submodels in the ensemble. The ensemble models will henceforth be referred to as ensemble linear regression (ELR), ensemble ridge regression (ERR), and ensemble neural network (ENN).
3. Third, because the optimal training set makeup depends on the design of the test set (Isidro y Sanchez & Akdemir [2021]), we developed a targeted training set for each location's genotypes. We optimized the ensemble by including only select locations in the ensemble. The optimization algorithm involved first acquiring prediction accuracies on each test location using the full ensemble containing all locations. Then, training locations were left out of the ensemble one by one, and predictions were made on a validation set. If the prediction accuracy increased when the location was left out, it was not included in the optimized ensemble. The optimized ensemble models will henceforth be referred to as optimized ensemble linear regression (OLR), optimized ensemble ridge regression (ORR), and optimized ensemble neural network (ONN).

### 2.4 Models

Across all three approaches shown in Figure 1, nine models were tested: SLR, ELR, OLR, SRR, ERR, ORR, SNN, ENN, and ONN. SLR, ELR, OLR, SRR, ERR, and ORR were implemented using the Python package scikit‐learn (Pedregosa et al., 2011). SNN, ENN, and ONN were implemented using the Python package PyTorch (Paszke et al., 2019). Code for the models can be found at https://github.com/McGillHaricots/peas‐andlove/tree/master/ML_Ensembles.

LR computes the ordinary least squares solution when using the coded genotypes to predict the value of the desired phenotype and can be defined as follows:

y=Xβ+ε,

where *y* is the response vector, *X* is an *n × m* matrix of SNP markers (*n* is the number of observations and *m* is 1 more than the number of different SNP markers), β is a vector of marker effects, or coefficients, and ε is the residual error. RR (McDonald, 2009) takes the same structural form but applies a penalty to the coefficients such that, instead of minimizing the sum of squared errors, the following cost function is minimized:

$${\left|\left|y-X\beta \right|\right|}^{2}+\lambda {\left|\left|\beta \right|\right|}^{2},$$

where the hyperparameter 𝜆 is the term that penalizes coefficients with a large magnitude. This results in a model regularized with the L2 norm, which uses the root of the sum of squares as the penalty to reduce large coefficients toward 0.

Neural networks use any number of nested functions to create a layered model that may discern nonlinear patterns not described by the linear models (Lecun et al., 2015). For each layer, *i*, denote the vector of node values by *a* ^(^ *^i^* ^)^ and the vector of biases by *b* ^(^ *^i^* ^)^. Let the weight between nodes *a* ^(^ *^i^* ^)^ *_k_* and *a* ^(^ *^i^* ^+ 1)^ *_j_* be denoted by *W* ^(^ *^i^* ^)^ *_jk_*, then the matrix of weights between layers *i* and *i* + 1 is given by *W* ^(^ *^i^* ^)^. So, *a* ^(^ *^i^* ^+ 1)^ can be computed from *a* ^(^ *^i^* ^)^ as follows:

$$a^{\left(i+1\right)}=F\left(a^{\left(i\right)}W^{\left(i\right)}+b^{\left(i+1\right)}\right),$$

where *F* is the activation function used in the network. The output layer of the network can be given in terms of the input layer, *a* ^(0)^, by repeated application of the equation above.

The weights and biases were updated during training to minimize the mean squared error loss function, using the Adam optimization algorithm (Adaptive Moment Estimation). This algorithm is a form of gradient descent that allows us to arrive at the ideal model weights that minimize our loss function (Kingma & Ba, 2014). The rectified linear unit activation function tends to yield the best model performance compared to other activation functions, so it was used as the activation function for this study (Perez‐Enciso & Zingaretti, 2019).

When implementing a neural network (NN), the first step is to tune hyperparameters including learning rate, batch size, the number of layers, and the width of each layer. To tune and select hyperparameters for the SNN, ENN, and ONN in each experiment, we performed threefold cross‐validation on the training set, which consisted of every environment except the one being used as the test set. For each experiment, the optimal hyperparameters were identified based on the average model performance across all three cross‐validation folds. The same hyperparameters were used for SNN, ENN, and ONN. We tested 300 combinations of learning rate (continuous uniform distribution on [10^−4^, $10^{0}$] in log 10 space); batch size (discrete uniform distribution on [2^2^,2^7^] in log 2 space), the number of layers (discrete uniform distribution on [3,8]), and the width of each layer (discrete uniform distribution on [3,100]) of the network were chosen using a random search. This was implemented using the NumPy, Scikit‐Learn, and PyTorch packages.

Montesinos‐Lopez et al. (2021) highlight that a large amount of data is required to train an accurate NN for GP; therefore, we modified our ensemble approach for the NN models as certain locations had as few as 30 observations. Bootstrap aggregation (bagging) was used to construct the training sets of the models comprising the ensemble (Breiman, 1996) instead of each training set being a single environment. In this case, bagging involves constructing the ensemble model training sets by random sampling (with replacement) of environments from the full training set. The number of samples used to make up the training set for each model was ultimately defined by the number of locations where the trait at hand was phenotyped. The number of models comprising the ensemble was determined through the hyperparameter tuning process.

The total loss of each NN ensemble was computed after each training epoch. Training stopped once the loss curve was both convex (decreasing at a slowing rate) and did not decrease by more than 5% between epochs. A visual representation of the training loss over epochs when training a single NN to predict SY can be viewed in Figure S1. However, the number of epochs was curtailed at 50 to reduce excessive computation on models that were training too slowly to be effective. Computation time was also significantly reduced by parallelization. The hyperparameter tuning within each test was parallelized using the Multiprocessing package within the Python standard library (Python Software Foundation, 2025); each test was again parallelized by being run as separate jobs in a high‐performance computing setting, as opposed to running each of the tests sequentially. The Digital Research Alliance of Canada provided high‐power computing resources.

### 2.5 BLUEs and heritability

First, we acquired means for each genotype in each year and each location from https://github.com/Alice‐MacQueen/CDBNgenomics/tree/master.

Then, we computed BLUEs for each genotype at each location, accounting for year. According to Holland and Piepho (2024), this approach is appropriate for unbalanced data like the data presented in this study. Using the R package *lme4* (Bates et al., 2015), we compute the BLUEs as follows:

$$Y_{ik}=\mu +G_{i}+S_{k}+\varepsilon_{ik},$$

where $Y_{ik}$ is the value of the *i*th genotype in the *k*th year, μ is the grand mean, $G_{i}$ is the fixed effect of the *i*th genotype, $\;S_{k}$ is the random effect of the *k*th year, and $\varepsilon_{ik}$ is the error term.

Using the variance components extracted from the model, the standard heritability in the broad sense was calculated for each individual location as follows (Covarrubias‐Pazaran, 2019):

$$\frac{\sigma_{g}^{2}}{\sigma_{g}^{2}+\sigma_{i}^{2}+\sigma_{e}^{2}},$$

where $\sigma_{g}^{2}$ is the genotypic variance, $\sigma_{i}^{2}$ is the variance accounted for by environment (in this case, the environment represents years within a location), and $\sigma_{e}^{2}$ is the residual variance.

### 2.6 Biplot analysis

To understand the relationship between the locations, we conducted a biplot analysis. We used the R package metan (Olivoto & Lúcio, 2020) to create a general linear model and visualize the effects of and relationships between different locations in the CDBN MET dataset. Following the example described in W. Yan et al. (2007), we conducted a genotype plus genotype‐by‐environment (GGE) analysis, using a model taking the form:

$$y_{ij}=u+a_{i}+B_{j}+\Phi_{ij},$$

where y is the phenotype of genotype *i* in environment *j*, u is the grand mean, *a* is the genotypic effect of the *ith* genotype, *B* is the environmental effect of the *j*th location, and $\Phi_{ij}$ is the G × E interaction effect. Data were environment‐centered so relationships among environments could be visualized (W. Yan & Tinker, 2006; W. Yan et al., 2007). Further information on interpreting biplots can be found in Hoyos‐Villegas et al. (2016) and Nachilima et al. (2021).

## 3 RESULTS

### 3.1 Model performance

**FIGURE 2:**
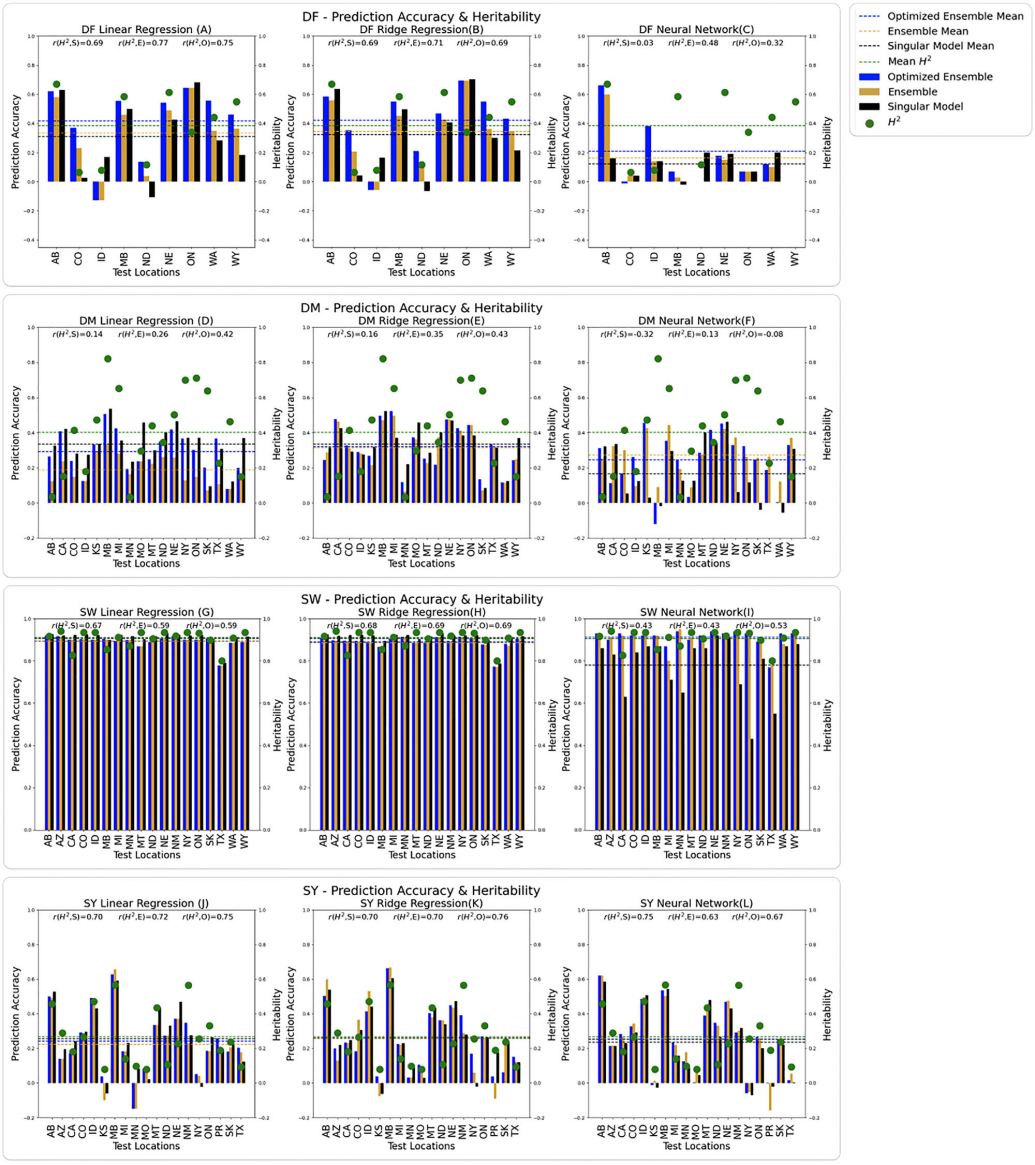
(A–C) Prediction accuracy for days to flowering (DF) for each test environment. (D–F) Prediction accuracy for days to maturity (DM). (G–I) Prediction accuracy for seed weight (SW). (J–L) Prediction accuracy for seed yield (SY). Prediction accuracy is measured as the Pearsons's correlation coefficient (PCC) between the true and estimated value. The left column shows linear regression. The black bar is singular linear regression (SLR), the blue bar is the optimized ensemble linear regression (OLR), and the orange bar is the bulk ensemble linear regression (ELR). The center column shows ridge regression. The black bar is singular ridge regression (SRR), the blue bar is the optimized ensemble ridge regression (ORR), and the orange bar is the bulk ensemble ridge regression (ERR). The right column shows neural networks. The black bat is the singular neural network (SNN), the blue bar is the optimized ensemble neural network (ONN), and the orange bar is the bulk ensemble neural network (ENN). The green dots show the broad sense heritability computed for each location. The correlation coefficient between broad‐sense heritability and model performance is indicated as the r value. The *r* values for the singular model, ensemble model, and optimized ensemble model are denoted *r*(*H* ^2^,S), *r*(*H* ^2^,E) and *r*(*H* ^2^,O), respectively.

The average model performance across all environments for DF using each model was 0.42 (OLR), 0.34 (ELR), 0.31 (SLR), 0.42 (ORR), 0.3 5 (ERR), 0.3 2 (SRR), 0.16 (ONN), 0.13 (ENN), and 0.131 (SNN). On average, the best performing models for DF were OLR (0.42) and ORR (0.42). When using LR (Figure 2A), RR (Figure 2B), and NNs (Figure 2C) to predict DF, the ensemble prediction accuracy overcame the singular approach in six out of nine environments and just three out of nine environments for NNs. OLR and ORR outperformed all three NN models in every environment. The highest observed accuracy for DF was 0.70 when using SRR to predict DF in ON. The lowest observed accuracy for DF was −0.13 when using OLR and ELR to predict DF in ID (Table S1).

The average model performance across all environments for DM using each model was 0.28 (OLR), 0.16 (ELR), 0.31 (SLR), 0.29 (ORR), 0.30 (ERR), 0.31 (SRR), 0.29 (ONN), 0.30 (ERR), and 0.18 (SNN). On average, the best performing model for DM was SLR and SRR (0.31). When using LR (Figure 2D), RR (Figure 2E), and NNs (Figure 2F) predict DM, the ensemble prediction accuracy overcame the singular approach in two out of 18 environments for LR, five out of 18 environments for RR, and seven out of 18 environments for NNs. The highest observed accuracy for DM was 0.47 when using ERR in MB, as well as when using SLR, ORR, ERR, and ARR in NE. The lowest observed accuracy for DM was −0.12 when using SNN in WA (Table S2).

The average model performance across all environments for SW using each model was 0.89 (OLR), 0.89 (ELR), 0.90 (SLR), 0.88 (ORR), 0.88 (ERR), 0.90 (SRR), 0.91 (ONN), 0.9 (ENN), and 0.787 (SNN). Overall, the best models for predicting SW SRR and ONN (0.91). When using LR (Figure 2G) to predict SW, the singular approach was ideal for all locations except for SK. When using RR (Figure 2H) to predict SW, the singular approach was ideal for all locations. Contrastingly, when using NNs to predict SW (Figure 2I), the ensemble prediction accuracy overcame the aggregate approach in nine out of 18 environments. The highest observed accuracy for SW was 0.95 when using ENN to predict SW in NE. The lowest observed accuracy for SW was 0.43 when using SNN in ON (Table S3).

The average model performance across all environments for SY using each model was 0.25 (OLR), 0.22 (ELR), 0.26 (SLR), 0.26 (ORR), 0.23 (ERR), 0.26 (SRR), 0.22 (ONN), 0.21 (ENN), and 0.20 (SNN). Overall, the best models for predicting SY were SLR, ORR, and SRR (0.26). When using LR (Figure 2J), RR (Figure 2K), and NNs (Figure 2L) to predict SY, the ensemble prediction accuracy overcame the aggregate approach in four out of 19 environments for LR, six out of 19 environments for LR, and seven out of 19 environments for NNs. The highest observed accuracy for SY was 0.48 when using ENN in MB and when using SNN in MT. The lowest observed accuracy for SY was ‐0.16 when using ENN in PR (Table S4).

An identical analysis was also conducted using BLUPs instead of BLUEs. We found a similar general trend in model accuracies between BLUPs and BLUEs and chose to present the results from BLUEs in this study because they account for the effect of year in each location, while the BLUPs did not. The accuracy results from the BLUPs experiment can be found in Tables S5–S8 for DF, DM, SW, and SY, respectively.

### 3.2 Heritability

Results from the heritability calculation for each location can be found in Table 1. The average heritability for each trait across locations was 0.38 (DF), 0.40 (DM), 0.91 (SW), and 0.31 (SY). For DF, the highest observed heritability was 0.67 in AB, and the lowest was 0.06 in CO. For DM, the highest observed heritability was 0.82 in MB, and the lowest observed heritability was 0.03 in MN. For SW, the highest observed heritability was 0.96 in NE, and the lowest observed heritability was 0.80 in TX. For SY, the highest observed heritability was 0.57 in MB, and the lowest observed accuracy was 0.08 in KS.

**TABLE 1:** Broad‐sense heritabilities per location.

Location	SY	DF	SW	DM
AB	0.46	0.67	0.92	0.04
AZ	0.29		0.94	–
CA	0.18	–	0.83	0.15
CO	0.26	0.06	0.95	0.41
ID	0.47	0.08	0.95	0.18
KS	0.08	–	–	0.47
MB	0.57	0.59	0.85	0.82
MI	0.14	–	0.91	0.65
MN	0.10	–	0.87	0.03
MO	0.08	–	–	0.30
MT	0.44	–	0.95	0.44
ND	0.11	0.12	0.91	0.35
NE	0.23	0.61	0.96	0.50
NM	0.56	–	0.92	–
NY	0.26	–	0.95	0.70
ON	0.33	0.34	0.93	0.71
PR	0.91	–	–	–
SK	0.24	–	0.90	0.64
TX	0.09	–	0.80	0.23
WA	–	0.44	0.91	0.46
WY	–	0.55	0.95	0.15
Mean	0.31	0.38	0.91	0.40

Abbreviations: DF, days to flowering; DM, days to maturity; SY, seed yield; SW, seed weight.

### 3.3 G × E interactions

**FIGURE 3:**
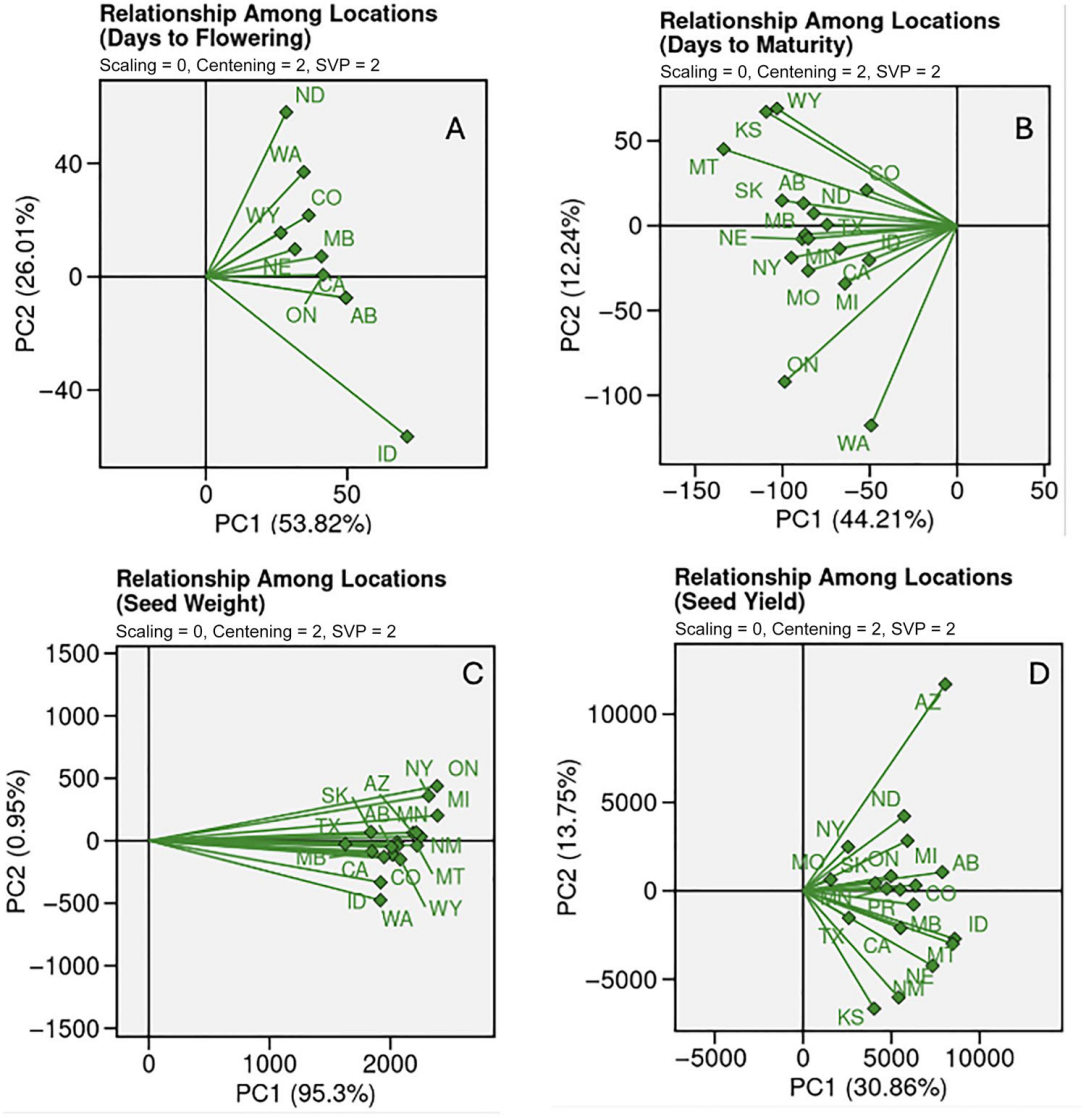
Biplot analysis of the observed genotypes in each location for days to flowering (A), days to maturity (B), seed weight (C), and seed yield (D). Longer vectors indicated locations with higher variance or discriminativeness. Shorter vectors indicate locations with lower variance, or low discriminativeness. The angle between the vectors indicates correlation between locations. PC, principal component; SVP, singular value partitioning.

The biplot analysis (Figure 3) revealed the relationship between the genotypes and the test locations. Vectors that are long indicate locations that are more discriminating among genotypes or that elicit higher variance among genotypes. Vectors that are short indicate locations that are less discriminating among locations or locations that do not reveal high variance among genotypes. The angle of the vectors describes the correlation among environments. Small angles between locations indicate highly correlated environments (Hoyos‐Villegas et al., 2016). For DF (Figure 3A), ID emerged as the most highly discriminating environment. NE and WY have short vectors and were therefore identified as less discriminating environments. CO, MB, CA, AB, ON, and WA were slightly more discriminating. For DM (Figure 3B), KS and WY were highly discriminating and highly correlated, as were ON and WA. MT was also relatively discriminating compared to AB, SK, MB, NE, NY, and MO, which were slightly less discriminating and CO, ND, TX, ID, MN, CA, and MI, which were the least discriminating. For SW (Figure 3C), the locations were notably more correlated compared to DF, DM, and SY. ON, MI, NM, MT, WY, and WA were the most discriminating environments. TX, MB, and CA showed notably low discriminativeness. For SY (Figure 3D), AZ was the most discriminating, followed by NE, KS, NM, MT, ID, and AB. MO, NY, TX, SK, PR, MI, MN, and ON were the least discriminating.

**FIGURE 4:**
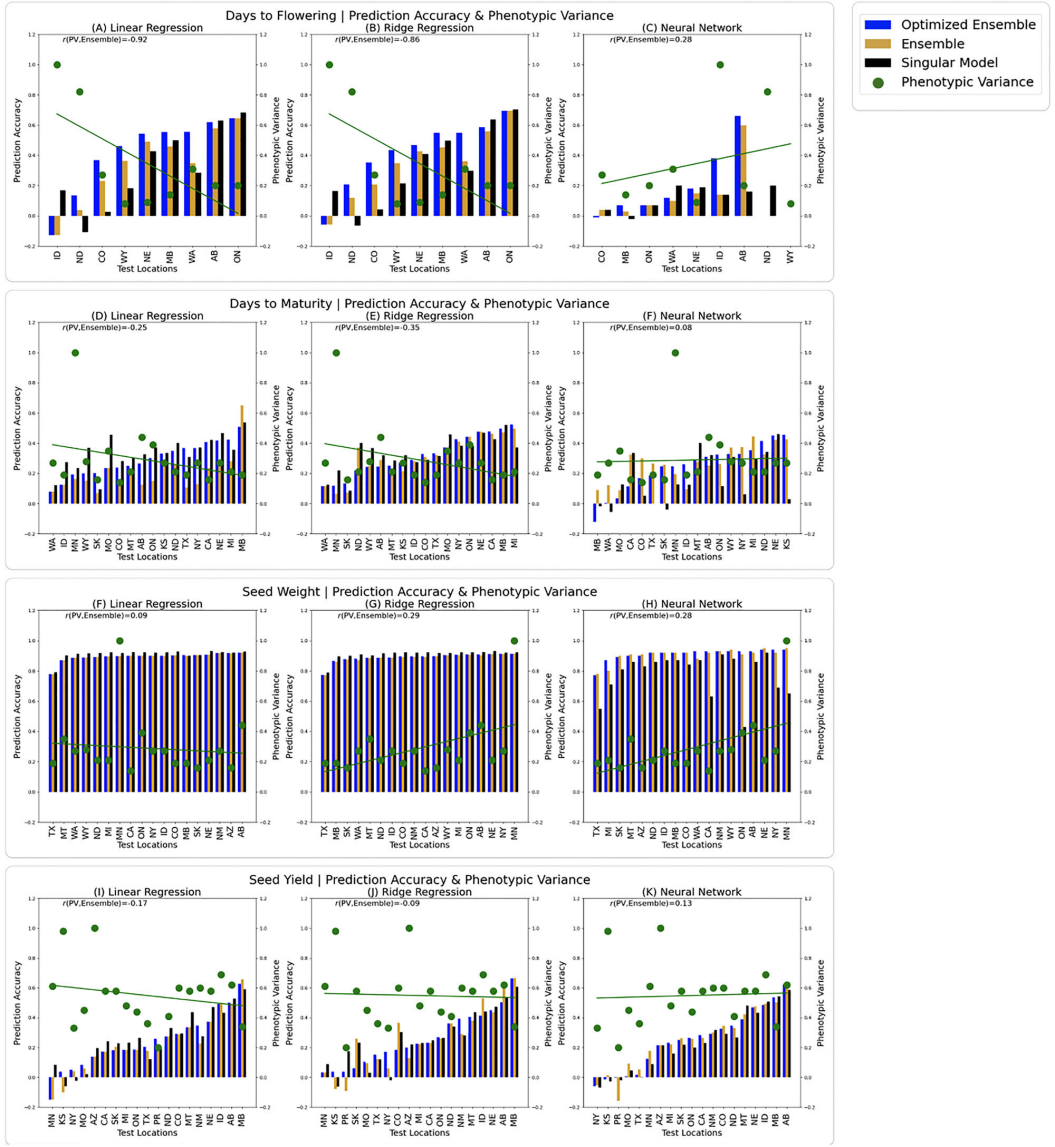
(A–C) Prediction accuracy for days to flowering (DF) for each test environment. (D–F) Prediction accuracy for days to maturity (DM). (G–I) Prediction accuracy for seed weight (SW). (J–L) Prediction accuracy for seed yield (SY). Prediction accuracy is measured as the Pearsons's correlation coefficient (PCC) between the true and estimated value for DF. The left column shows linear regression. The black line is singular linear regression (SLR), the blue bar is the optimized ensemble linear regression (OLR), and the orange bar is the bulk ensemble linear regression (ELR). The center column shows ridge regression. The black line is singular ridge regression (SRR), the blue bar is the optimized ensemble (ORR), and the orange bar is the bulk ensemble linear regression (ERR). The right column shows neural networks. The black line is the singular neural network (SNN), the blue bar is the optimized ensemble neural network (ONN), and the orange bar is the bulk ensemble neural network (ENN). The green dots show the phenotypic variance computed for each location. Correlation between the model prediction accuracy and the phenotypic variance is incdicated at the top of each graph as *r*(PV,S), indicating the correlation between the phenotypic variance and the singular model, *r*(PV,E), indicating the correlation between the phenotypic variance and the ensemble model, and *r*(PV,O), indicating the correlation between the phenotypic variance and the optimized ensemble model.

In addition to conducting the biplot analysis, we calculated the phenotypic variance for each location and plotted the phenotypic variance against model performance (Figure 4). All values for phenotypic variance, broad sense heritability, genetic variance, and residual variance can be seen in Tables S9 (DF), S10 (DM), S11 (SW), and S12 (SY).

Figure 4 shows prediction accuracy and model performance for each model and each location. The locations along the *X*‐axis are arranged according to increasing ensemble model performance to illustrate the inverse relationship between phenotypic variance and ensemble model accuracy. Figure 4A,B,C shows prediction accuracy for DF across all models. It indicates that for all LR models (4A) and RR (4B), the singular model approach worked best at the location where phenotypic variance was highest (ID). Figure 4D,E,F shows prediction accuracy for DM across all models. Where phenotypic variance is highest (WA, ID, MN, and WY), the singular approach is best, while where phenotypic variance is lowest (MB, MI, CO, and CA), the ensemble approach is best. Figure 4G,H,I shows prediction accuracy for SW across all models. The inverse relationship between phenotypic variance and ensemble model performance was not observed when predicting SY. However, this trend was pronounced in SY. Figure 4J,K,L indicates the relationship between model performance and phenotypic variance for SY. For the locations with the lowest variance (MB, NY, PR, and AB), the ensemble model had higher accuracy than the singular model.

## 4 DISCUSSION

We found that when estimating the breeding value of genotypes in an unseen environment, the ensemble model approach was better than the singular model if the test location elicits low phenotypic variance, or low separation among genotypes (Figure 3). This effect was more pronounced when the training samples used to train the ensemble model were optimized to maximize prediction accuracy (i.e., the optimized ensemble). To exemplify this effect, we can consider SY. According to the biplot analysis, environments with low discriminativeness included NY, MO, SK, and TX. As a result, the optimized ensemble approach was ideal for these environments. Contrastingly, the most discriminating (highest variance) locations were AZ, NE, and MT. When predicting breeding values in these locations, the singular model approach was best.

This finding can be explained in terms of the “variance/bias tradeoff.” According to Briscoe and Feldman (2011), the bias‐variance trade‐off can be conceptualized as the granularity with which the model describes the training data. A model that describes the training data with extreme granularity will have a high level of variance but may also pick up on randomness or noise in the data. We may also describe a very granular, high‐variance model as an overfitted model. If we train a model so that it is no longer overfitted, we will reduce model complexity and introduce a higher level of bias in return. A highly biased model is more constrained, places expectations on the data, and is minimally complex. It is a well‐known feature of ensembles (when constructed using small subsets of the full training set to train the individual submodels in the ensemble) that variance decreases without introducing more bias (Miyenye & Sun, 2022). In other words, ensembles allow us to bypass the bias/variance trade‐off. If we consider our four traits (DF, DM, SW, and SY), our three models (LR, RR, and NN), and our three training approaches (singular, ensemble, and optimized ensemble), we can conclude that the optimized ensemble approach using RR as the model was a top performer, on average, for all traits. This is likely because a RR model is regularized, constraining the coefficients and making the model more transferable by preventing overfitting. This beneficial effect is exacerbated when we create an ensemble of RR models working together to produce a prediction, because by averaging all the submodels in the ensemble, we further reduce model variance (without increasing bias), making the model more transferable to unseen data. While this was the case on average, there were certain locations where this effect was not favorable, as discussed previously, because these locations exhibit high separation (variance) among genotypes. In this case, a model with higher variance is best to match the high variance in the test environment.

We also found that when using NNs, the ensemble approach overcame the singular model approach in seven out of 18 environments (DM), nine out of 18 environments (SW), and seven out of 19 environments (SY). This suggests that when it comes to using NNs for GP, data volume is not the only consideration. If a breeder has accumulated a large volume of data and wishes to use a nonparametric model such as NNs for GP, it may be ideal to use these data to train an ensemble model (as opposed to a singular model) for optimizing prediction accuracy. The ensemble approach is especially useful when the base model is unstable, which is the case when using NNs for GP. Further, we found that the highest observed accuracy across the entire study was achieved when predicting SW using ENN in MN (0.95). We found that on average, the ensemble models performed better than the singular models for DF (all models), DM (NNs), SW (NNs), and SY (NNs). This suggests that NNs can be a valuable tool for plant breeders, but linear and RR remain more robust across traits and environments.

### 4.1 Utility of ensemble approaches

As it allows for the bias/variance trade‐off to be mitigated, ensemble approaches are a useful tool and have been previously investigated in the GP approach. This effect was previously investigated by Fradgley et al. (2023) who concluded that using an ensemble approach may help to stabilize gains across multiple traits in a closed gene pool. They used a random forest model, which is one of the most implemented ensemble models, in combination with a multi‐trait ensemble to achieve gain in yield and other important traits in a simulation study. Liang et al. (2021), Sun et al. (2012), and Abdollahi‐Arpanahi et al. (2020) found ensemble approaches that showed high prediction accuracy in cattle populations. Further, Ogutu et al. (2011) found that an ensemble approach called boosting performed better than other methods on a simulated maize dataset. Fernandez‐Gonzalez et al. (2024) also used an ensemble approach called gradient boosting (which fits an ensemble of weak learners on model residuals) and found that it could effectively quantify nonadditive effects when predicting the value of sunflower hybrids. They used criteria of diversity and relatedness to the test set to determine which data from which years should be used to compose the ideal training set. We also used an optimization algorithm to compose the test set, but did so according to the informativeness of locations in order to make our model more robust. In general, this indicates training set optimization algorithms are key when working with a large training set. Banerjee et al. (2020) also found that using an ensemble approach made for a more robust model; however, they used a simple “bagging” approach, which differed from our approach for LR and RR in that the training sets for each submodel in the ensemble were not randomly sampled but were instead made up of datasets from a given location. They highlight the utility of an ensemble approach to mitigate the variance/bias trade‐off, which we also found in our study when making predictions on genotypes in low‐variance environments.

Ensembles may be a useful way for working with large MET datasets. J. Yan et al. (2021) implemented an ensemble approach called LightGBM for predicting days to tasseling, plant height, and ear weight in maize using a multi‐year dataset. We also found that turning multi‐environment data into an ensemble model was useful for increasing prediction accuracy. Notably, prediction accuracy increased from 0.03 to 0.37 when the ensemble approach was used compared to the aggregate approach to predict DF in CO (OLR), from 0.37 to 0.52 when predicting DM in MI (ORR), from 0.45 to 0.93 when used to predict SW in ON (ONN), and from −0.02 to 0.17 when used to predict SY in NY (ORR). Westhues et al. (2021) also investigated the utility of machine learning models when predicting the performance of maize genotypes in new environments. They found that for certain traits, ensemble models such as decision tree‐based gradient boosting were superior to other regression approaches that incorporate G × E as a model covariate. They note that a benefit of this approach is the interpretability of the model, which is also a benefit of the across‐environment LR and RR ensembles tested in this study.

In general, ensembles are made up of decision trees (called random forests) or linear models trained to reduce residuals and boost performance (called gradient boosting). It is less common to train an ensemble of NNs, but our results indicate that NN ensembles may be able to increase the performance of NNs. A review by Ganaie et al. (2022) supports the idea that these “deep ensembles” may allow us to reap both (1) benefits of NNs in terms of their ability to pick up nonlinear patterns and (2) ability of an ensemble to boost accuracy and sidestep the bias/variance tradeoff. In addition, using an ensemble can prevent the model from getting stuck at a local minimum when training and increase the likelihood of arriving at the global minimum of the loss function. A notable case from our study is the prediction of DM in KS and in SK. ONN performed better than all other models, and further, the margin of improvement from the singular model to the ensemble model was greatest for the NN with an increase from 0.03 to 0.46 in KS and from −0.04 to 0.25 in SK. In these cases, NN may have been deemed inappropriate for the task at hand. However, the NN outperformed other approaches when used in an ensemble approach. When using NNs, computational intensity can be challenging, but we found that accessing high‐performance computing and parallelization made the experiment computationally feasible.

In general, the linear models (LR and RR) performed better than NN which is in accord with the literature. In their review, Montesinos‐Lopez et al. (2021) indicate that while they do pick up nonlinear patterns, there is no clear advantage to using deep NNs. We observed one exception to this pattern in which the ONN model performed better than all other models when predicting SW in MN with an accuracy of 0.95, when used to predict SY in AB (0.62) and when predicting DM in KS (0.46) and SK (0.25). This indicates that there may be cases in which the ability of NN to pick up nonlinear interactions in the genome may be able to recover accuracy that is lost when only considering linear additive effects. However, for the majority of cases, the highest accuracy across all locations was achieved when using LR and RR.

### 4.2 Heritability variation among traits

Highly heritable traits tend to correlate with higher prediction accuracy, described by Kaler et al. (2022) and Zhang et al. (2017). Accordingly, the average prediction accuracies for each trait were 0.25 (SY), 0.27 (DM), 0.28 (DF), and 0.89 (SW). The average heritability for these traits, respectively, were 0.31, 0.40, 0.38, and 0.91, exemplifying the correlation between heritability and prediction accuracy. These heritability values are comparable to those computed by MacQueen et al. (2020). They found that narrow‐sense heritability was 0.43 for DF, 0.40 for DM, 0.69 for SW, and 0.19 for SY. We can observe in Figure 2 this general correlation between prediction accuracy and marker‐based heritability for each test location. The high prediction accuracy for SW may also be explained by its genetic architecture. It is characterized by quantitative inheritance; however, it has been shown to lack transgressive segregation, and has also been shown, in certain populations, to have QTL that cluster on one chromosome (Geravandi et al., 2020). The presence of fewer linkage groups controlling SW may have contributed to this high accuracy because the model accuracy did not depend on many small effects or epistatic effects. Our narrow‐sense heritability calculations revealed that the heritability of SW was high. The high accuracy for SW may also be due to the high correlation between each of the locations (Figure 3), which led to an overfit model.

Considering that heritability is the proportion of phenotypic variance due to variation in the genotypes for a target trait, we could argue that GS will not be superior to phenotypic selection unless prediction accuracy overcomes heritability. For DF, this occurred in CO, ID, ND, ON, and WA. For DM, this occurred in AB, CA, ID, MN, MO, ND, TX, and WY. For SW, this occurred in CA, MB, and MN. For SY, this occurred in all AB, CA, CO, ID, MB, MI, MO, ND, NE, PR, and TX. This suggests that unless a breeder is observing exceptionally high heritability for a trait in their program, GS may be better for making selections than phenotypic selection (PS), but more investigation would be needed to confirm this.

There were also a handful of unique cases where the use of the optimized ensemble approach permitted prediction accuracy to overcome heritability. For example, predicting DF in CO using LR or predicting SY in MB using LR. These locations (CO and MB) were moderately low variance according to the biplot, so it is not a surprise the ensemble approach performed exceptionally well there. On average, the performance certainly was able to overcome average heritability for all traits (Figure 2). For DF, these models were ELR and ERR. For SW this was the case for the ENN. This supports the idea that the merit of using GS over PS depends on many interacting factors including trait heritability, model, training set construction, and test location.

### 4.3 Biplot analyses to assist GS implementation

A trend emerged in the biplots explaining why the ensemble (both optimized and un‐optimized) approach increased prediction accuracy in some environments and decreased prediction accuracy in others. In general, the locations with a short vector in the biplot (indicating a low level of discriminativeness compared to the other environments) benefitted from the ensemble approach. The ensemble approach resulted in higher accuracy than the aggregated LR. Using SY as an example, the location with the longest vector and highest discriminativeness was AZ, and the singular approach outperformed the ensemble approach here, whereas the ensemble approach was best in TX, PR, and MO, all of which had short vectors on the biplot (see Figure 2J,K; Table S4). DM also provides a clear example of this trend where the locations with the longest vectors in Figure 3 (WA, WY, MT, and ON) also favored the singular approach while the short‐vector locations (ID, TX, and CO) favored the ensemble approach (see Figure 2D,E; Table S2). While there are some exceptions to this trend, the negative correlation coefficient between the ensemble approach and the phenotypic variance at a given location supports this general trend. This indicates that for an environment where discriminativeness between genotypes is low (low phenotypic variance), employing the ensemble approach will allow breeders to better predict how genotypes will perform in that environment. This is because when we average across models, we end up with a closer fit (lower variance). Typically, decreasing model variance results in an increase in model bias. However, a benefit of the ensemble approach is that we can create a tighter model fit without creating a highly biased model. For those environments where discrimination is high, a very tight fit (low variance) is not desirable. If an environment is very discriminative (high phenotypic variance), a model that has higher variance would be a better fit. This explains why a single aggregate model results in higher accuracy for these environments.

Notably, the accuracy for SW was high across all locations. This may be explained by the biplot analysis (Figure 3C), which shows that the locations were more correlated for SW than for the other three traits. Therefore, when we predict for a single location, there is a high correlation between the GEBVs and the BVs. The training phenotypes resemble the testing phenotypes regardless of which location is being used for testing. A similar trend was observed for DF. The locations ID and ND showed low correlation with the other environments (Figure 3A) indicated by the vectors with a larger angle from the other environments. The lowest prediction accuracy was observed for DF in these environments. Similarly, for SY, KS saw lower prediction accuracy than all other environments and had the most obtuse angle from the center of the plot compared to the other locations.

It is important to note that breeders may not be aware of the nature of the environments that they are working with. We did find that for certain trait/model combinations, there was a phenotypic variance threshold for locations below which the ensemble approach was ideal. To quantify this threshold, we computed the proportion of variance present at this threshold compared to the maximum level of variance observed in the trial. We found that the average threshold proportion was 14.51% of maximum observed variance across locations. Hence, even if a breeder has not conducted a complete GGE analysis, historical data in the breeding program could be leveraged to determine what level of phenotypic variance is present at their location, compared to other locations in which the same trait was phenotyped. Ideally, GP model optimization should go hand‐in‐hand with analyses of test environments. Even if a MET is not available for training, a simple training set can still be used to train an ensemble model by using the bagging approach. Bagging stands for “bootstrap aggregating” and involves repeated sampling a random subset of test observations with replacement. These sampled sets can be used to train the submodels comprising the ensemble. More information on bagging can be found in Breiman (1996).

### 4.4 Using accumulated data

Robust prediction accuracy can be achieved when data across time and locations is accumulated by breeders. Empirical validation of GS will be an ongoing effort as breeders accumulate data for their training populations over time. The CDBN MET dataset shows us that breeding programs can collaborate to produce a robust training set. Many programs use similar checks and parents, so over time programs can pool their data to generate a large, relatively connected dataset to be used for GP model training. Similarly, Sneller et al. (2021) put forth the idea of Consortium‐based breeding in which programs collaborate to share resources, especially because there tends to be overlap in germplasm between programs of a specific crop. They encourage germplasm sharing and a diffusion of phenotyping across programs to implement robust sparse testing. We propose an additional component to consortium breeding in which large GS training sets are shared among programs and optimized for individual programs depending on their location.

Atanda et al. (2021) also leveraged historic data to optimize training set construction in a MET scenario. They found that prediction accuracy was higher when phenotypic data were collected for target genotypes in target locations so that genetic information is equally represented across environments. Our results are in accord with their conclusion that care should be taken to select the most informative data from a historical dataset, instead of using all data, or a random subset under the assumption that relatedness in the program is high and, therefore, all data points are appropriate for training. Predicting previously observed genotypes in unobserved environments is significant because it will allow breeders to expand the number of genotypes included in a trial, assessing a larger array of allelic effects and increasing the intensity of selection in their program without expanding the space and labor required for phenotyping. Bernal‐Vasquez et al. (2017) point out that while it is simpler for breeders to implement a single‐year model, there is a benefit to including several years’ worth of data for optimizing the prediction accuracy by better approximating genetic effects. Keller et al. (2020) made predictions for common bean genotypes in future seasons, which was similar to our approach of making predictions in unobserved locations. They found that including data from all available trials increased accuracy, favoring testing in many environments over testing many replications in one environment. Keller et al. (2020) accumulated data over time, which is equivalent to the SLR, SRR, and SNN models. We found that depending on the test location, accumulating data over time into one aggregate model may be ideal; however, in some cases, using historical data to create an ensemble model may result in higher prediction accuracies.

## 5 CONCLUSION

In this study, we used the CDBN MET dataset to assess three different ways that multi‐environment data can be used to construct a GP model: (1) aggregate all data into a singular model, (2) create an ensemble model using all data, and (3) create an ensemble model using an optimized selection of the available data. For those locations where variance among genotypes is high, aggregating all available data was a better approach than training an ensemble. Conversely, for those locations where variance among genotypes is low, creating an ensemble model is ideal. We found that the MET data were useful for making predictions across locations, and GGE analysis is a tool to guide model training and finding approaches for training set construction. Transferring a given prediction model across locations may result in disparate accuracies even on previously observed genotypes, but optimizing the locations used for training or choosing to train an aggregate versus ensemble model may mitigate this disparity in performance across locations. For models with low performance in general, such as NNs, the ensemble approach can be tested as a method for increasing prediction accuracy. Because pooled data from the CDBN MET were able to produce reasonable prediction accuracies across many locations, this study reveals that breeding program collaboration can be a useful way to bypass the data volume bottleneck for machine learning GP models. In doing so, breeders may be able to implement GS for traits with low heritability to increase selection accuracy and increase the rate of genetic gain in their programs.

## AUTHOR CONTRIBUTIONS

**Isabella Chiaravallotti**: Conceptualization; data curation; formal analysis; investigation; methodology; validation; visualization; writing—original draft; writing—review and editing. **Owen Pauptit**: Data curation; formal analysis; methodology. **Valerio Hoyos‐Villegas**: Conceptualization; methodology; project administration; resources; supervision; writing—review and editing.

## CONFLICT OF INTEREST STATEMENT

The authors declare no conflicts of interest.

## Supporting information




**Supplemental Figure 1**: Training loss for a neural network. The y‐axis indicates the value for the mean squared error loss function at a given iteration, indicated on the x‐axis. The model has converged and training is complete either (1) when the loss function no longer changes beyond a given threshold, (2) or when the number of iterations is complete. In Figure 1, training was considered complete by the time 500 iterations had run. When we ran our experiments, we set the maximum number of iterations to 50 due to computational time, and the assumption below that 50 iterations is sufficient for minimizing loss.

**Supplemental Table 1**: Prediction accuracy for days to flowering for each test location. The table includes the Pearson's correlation coefficient between true and estimated values when using optimized the ensemble, bulk ensemble, and aggregate model for linear regression, ridge regression, and neural network.
**Supplemental Table 2**: Prediction accuracy for days to maturity for each test location. The table includes the Pearson's correlation coefficient between true and estimated values when using optimized the ensemble, bulk ensemble, and aggregate model for linear regression, ridge regression, and neural network.
**Supplemental Table 3**: Prediction accuracy for seed weight for each test location. The table includes the Pearson's correlation coefficient between true and estimated values when using optimized the ensemble, bulk ensemble, and aggregate model for linear regression, ridge regression, and neural network.
**Supplemental Table 4**: Prediction accuracy for seed yield for each test location. The table includes the Pearson's correlation coefficient between true and estimated values when using optimized the ensemble, bulk ensemble, and aggregate model for linear regression, ridge regression, and neural network.
Supplemental Table 5: Prediction accuracy for days to flowering for each test location using BLUPs. The table includes the Pearson's Correlation Coefficient between true and estimated values when using optimized the ensemble, bulk ensemble, and aggregate model for linear regression, ridge regression, and neural network
Supplemental Table 6: Prediction accuracy for days to maturity for each test location using BLUPs. The table includes the Pearson's Correlation Coefficient between true and estimated values when using optimized the ensemble, bulk ensemble, and aggregate model for linear regression, ridge regression, and neural network.
Supplemental Table 7: Prediction accuracy for seed weight for each test location using BLUPs. The table includes the Pearson's Correlation Coefficient between true and estimated values when using optimized the ensemble, bulk ensemble, and aggregate model for linear regression, ridge regression, and neural network.
Supplemental Table 8: Prediction accuracy for seed yield for each test location using BLUPs. The table includes the Pearson's Correlation Coefficient between true and estimated values when using optimized the ensemble, bulk ensemble, and aggregate model for linear regression, ridge regression, and neural network.
Supplemental Table 9: For days to flowering: broad‐sense heritability (*H* ^2^), genetic variance (GV), phenotypic variance (PV), residual variance (RV) and the proportion of the maximum observed variance observed across all locations present at each location (Percent Var)
Supplemental Table 10: For days to maturity: phenotypic variance (pv), borad‐sense heritability (*H* ^2^), additive genetic variance (va), error variance (ve) and the proportion of the maximum observed across all locations present at each location (PercentVar)
Supplemental Table 11: For seed weight: phenotypic variance (pv), broad‐sense heritability (*H* ^2^), additive genetic variance (va), error variance (ve) and the proportion of the maximum observed across all locations present at each location (PercentVar)
Supplemental Table 12: For seed yield: phenotypic variance (pv), broad‐sense heritability (*H* ^2^), additive genetic variance (va), error variance (ve) and the proportion of the maximum observed across all locations present at each location (PercentVar).

## Data Availability

The code for the cross‐validation experiment can be found at https://github.com/McGillHaricots/peas‐andlove/tree/master/ML_Ensembles. The raw data for the CDBN MET can be found at https://github.com/McGillHaricots/CDBN‐GeneticGain and https://github.com/Alice‐MacQueen/CDBNgenomics.
