# Supplementary material for: Environment ensemble models for genomic prediction in common bean (Phaseolus vulgaris L.)
Source: Plant Genome. 2025 Jun 12;18(2):e70057. doi: 10.1002/tpg2.70057 (PMC12159719; doi:10.1002/tpg2.70057)
Supplement: Supplementary file 1 [file TPG2-18-e70057-s002.docx]

**Supplemental Tables**

**Supplemental Table 1:** Prediction accuracy for days to flowering for each test location. The table includes the Pearson’s Correlation Coefficient between true and estimated values when using optimized the ensemble, bulk ensemble, and aggregate model for linear regression, ridge regression, and neural network.

| **Prediction Accuracy for Each Location (Days to Flowering)** | | | | | | | | | |
| --- | --- | --- | --- | --- | --- | --- | --- | --- | --- |
|  | **Linear Regression** | | | **Ridge Regression** | | | **Neural Network** | | |
| **Location** | **OLR** | **ELR** | **SLR** | **ORR** | **ERR** | **SRR** | **ONN** | **ENN** | **SNN** |
| AB | 0.62 | 0.58 | 0.63 | 0.58 | 0.56 | 0.64 | 0.66 | 0.60 | 0.16 |
| CO | 0.37 | 0.23 | 0.03 | 0.35 | 0.21 | 0.04 | -0.01 | 0.04 | 0.04 |
| ID | -0.13 | -0.13 | 0.17 | -0.06 | -0.06 | 0.16 | 0.38 | 0.14 | 0.14 |
| MB | 0.55 | 0.46 | 0.50 | 0.55 | 0.45 | 0.50 | 0.07 | 0.03 | -0.02 |
| ND | 0.14 | 0.04 | -0.11 | 0.21 | 0.12 | -0.06 | 0.00 | 0.00 | 0.20 |
| NE | 0.54 | 0.49 | 0.43 | 0.47 | 0.43 | 0.41 | 0.18 | 0.15 | 0.19 |
| ON | 0.64 | 0.64 | 0.68 | 0.69 | 0.69 | 0.70 | 0.07 | 0.07 | 0.07 |
| WA | 0.56 | 0.35 | 0.28 | 0.55 | 0.36 | 0.30 | 0.12 | 0.10 | 0.20 |
| WY | 0.46 | 0.36 | 0.18 | 0.43 | 0.35 | 0.21 | 0.00 | 0.00 | 0.00 |
| **Mean** | **0.42** | **0.34** | **0.31** | **0.42** | **0.35** | **0.32** | **0.16** | **0.13** | **0.11** |

**Supplemental Table 2:** Prediction accuracy for days to maturity for each test location. The table includes the Pearson’s Correlation Coefficient between true and estimated values when using optimized the ensemble, bulk ensemble, and aggregate model for linear regression, ridge regression, and neural network.

| **Prediction Accuracy for Each Location (Days to Maturity)** | | | | | | | | | |
| --- | --- | --- | --- | --- | --- | --- | --- | --- | --- |
|  | **Linear Regression** | | | **Ridge Regression** | | | **Neural Network** | | |
| **Location** | **OLR** | **ELR** | **SLR** | **ORR** | **ERR** | **SRR** | **ONN** | **ENN** | **SNN** |
| AB | 0.92 | 0.92 | 0.93 | 0.91 | 0.90 | 0.93 | 0.93 | 0.92 | 0.86 |
| AZ | 0.92 | 0.92 | 0.92 | 0.90 | 0.90 | 0.92 | 0.90 | 0.91 | 0.83 |
| CA | 0.90 | 0.90 | 0.92 | 0.90 | 0.89 | 0.92 | 0.93 | 0.92 | 0.63 |
| CO | 0.90 | 0.90 | 0.93 | 0.89 | 0.89 | 0.92 | 0.92 | 0.92 | 0.84 |
| ID | 0.90 | 0.90 | 0.92 | 0.89 | 0.89 | 0.92 | 0.92 | 0.92 | 0.87 |
| MB | 0.90 | 0.90 | 0.90 | 0.87 | 0.86 | 0.89 | 0.92 | 0.92 | 0.87 |
| MI | 0.89 | 0.89 | 0.92 | 0.91 | 0.91 | 0.92 | 0.87 | 0.80 | 0.71 |
| MN | 0.90 | 0.90 | 0.92 | 0.91 | 0.91 | 0.92 | 0.94 | 0.95 | 0.65 |
| MT | 0.87 | 0.87 | 0.90 | 0.89 | 0.88 | 0.90 | 0.90 | 0.91 | 0.86 |
| ND | 0.89 | 0.89 | 0.92 | 0.89 | 0.89 | 0.92 | 0.92 | 0.92 | 0.86 |
| NE | 0.91 | 0.91 | 0.93 | 0.91 | 0.91 | 0.93 | 0.94 | 0.95 | 0.92 |
| NM | 0.92 | 0.92 | 0.92 | 0.89 | 0.89 | 0.92 | 0.93 | 0.93 | 0.91 |
| NY | 0.90 | 0.90 | 0.92 | 0.91 | 0.91 | 0.92 | 0.94 | 0.92 | 0.69 |
| ON | 0.90 | 0.90 | 0.92 | 0.91 | 0.91 | 0.92 | 0.93 | 0.91 | 0.43 |
| SK | 0.90 | 0.91 | 0.90 | 0.88 | 0.88 | 0.90 | 0.89 | 0.90 | 0.81 |
| TX | 0.78 | 0.78 | 0.79 | 0.77 | 0.77 | 0.79 | 0.77 | 0.78 | 0.55 |
| WA | 0.89 | 0.89 | 0.91 | 0.88 | 0.87 | 0.91 | 0.93 | 0.88 | 0.87 |
| WY | 0.89 | 0.89 | 0.92 | 0.90 | 0.90 | 0.92 | 0.93 | 0.94 | 0.88 |
| **Mean** | **0.89** | **0.89** | **0.90** | **0.88** | **0.88** | **0.90** | **0.91** | **0.90** | **0.77** |

**Supplemental Table 3:** Prediction accuracy for seed weight for each test location. The table includes the Pearson’s Correlation Coefficient between true and estimated values when using optimized the ensemble, bulk ensemble, and aggregate model for linear regression, ridge regression, and neural network.

| **Prediction Accuracy for Each Location (Seed Weight)** | | | | | | | | | |
| --- | --- | --- | --- | --- | --- | --- | --- | --- | --- |
|  | **Linear Regression** | | | **Ridge Regression** | | | **Neural Network** | | |
| **Location** | **OLR** | **ELR** | **SLR** | **ORR** | **ERR** | **SRR** | **ONN** | **ENN** | **NN** |
| AB | 0.92 | 0.92 | 0.93 | 0.91 | 0.90 | 0.93 | 0.93 | 0.92 | 0.86 |
| AZ | 0.92 | 0.92 | 0.92 | 0.90 | 0.90 | 0.92 | 0.90 | 0.91 | 0.83 |
| CA | 0.90 | 0.90 | 0.92 | 0.90 | 0.89 | 0.92 | 0.93 | 0.92 | 0.63 |
| CO | 0.90 | 0.90 | 0.93 | 0.89 | 0.89 | 0.92 | 0.92 | 0.92 | 0.84 |
| ID | 0.90 | 0.90 | 0.92 | 0.89 | 0.89 | 0.92 | 0.92 | 0.92 | 0.87 |
| MB | 0.90 | 0.90 | 0.90 | 0.87 | 0.86 | 0.89 | 0.92 | 0.92 | 0.87 |
| MI | 0.89 | 0.89 | 0.92 | 0.91 | 0.91 | 0.92 | 0.87 | 0.80 | 0.71 |
| MN | 0.90 | 0.90 | 0.92 | 0.91 | 0.91 | 0.92 | 0.94 | 0.95 | 0.65 |
| MT | 0.87 | 0.87 | 0.90 | 0.89 | 0.88 | 0.90 | 0.90 | 0.91 | 0.86 |
| ND | 0.89 | 0.89 | 0.92 | 0.89 | 0.89 | 0.92 | 0.92 | 0.92 | 0.86 |
| NE | 0.91 | 0.91 | 0.93 | 0.91 | 0.91 | 0.93 | 0.94 | 0.95 | 0.92 |
| NM | 0.92 | 0.92 | 0.92 | 0.89 | 0.89 | 0.92 | 0.93 | 0.93 | 0.91 |
| NY | 0.90 | 0.90 | 0.92 | 0.91 | 0.91 | 0.92 | 0.94 | 0.92 | 0.69 |
| ON | 0.90 | 0.90 | 0.92 | 0.91 | 0.91 | 0.92 | 0.93 | 0.91 | 0.43 |
| SK | 0.90 | 0.91 | 0.90 | 0.88 | 0.88 | 0.90 | 0.89 | 0.90 | 0.81 |
| TX | 0.78 | 0.78 | 0.79 | 0.77 | 0.77 | 0.79 | 0.77 | 0.78 | 0.55 |
| WA | 0.89 | 0.89 | 0.91 | 0.88 | 0.87 | 0.91 | 0.93 | 0.88 | 0.87 |
| WY | 0.89 | 0.89 | 0.92 | 0.90 | 0.90 | 0.92 | 0.93 | 0.94 | 0.88 |
| **Mean** | **0.89** | **0.89** | **0.91** | **0.89** | **0.89** | **0.91** | **0.91** | **0.91** | **0.78** |

**Supplemental Table 4**: Prediction accuracy for seed yield for each test location. The table includes the Pearson’s Correlation Coefficient between true and estimated values when using optimized the ensemble, bulk ensemble, and aggregate model for linear regression, ridge regression, and neural network.

| **Prediction Accuracy for Each Location (Seed Yield)** | | | | | | | | | |
| --- | --- | --- | --- | --- | --- | --- | --- | --- | --- |
|  | **Linear Regression** | | | **Ridge Regression** | | | **Neural Network** | | |
| **Location** | **OLR** | **ELR** | **SLR** | **ORR** | **ERR** | **SRR** | **ONN** | **ENN** | **SNN** |
| AB | 0.50 | 0.49 | 0.53 | 0.50 | 0.60 | 0.54 | 0.62 | 0.62 | 0.59 |
| AZ | 0.14 | 0.14 | 0.20 | 0.20 | 0.13 | 0.22 | 0.21 | 0.22 | 0.21 |
| CA | 0.17 | 0.17 | 0.24 | 0.23 | 0.23 | 0.25 | 0.28 | 0.26 | 0.23 |
| CO | 0.29 | 0.29 | 0.29 | 0.18 | 0.37 | 0.30 | 0.33 | 0.34 | 0.29 |
| ID | 0.49 | 0.49 | 0.43 | 0.41 | 0.53 | 0.44 | 0.49 | 0.50 | 0.51 |
| KS | 0.04 | -0.10 | -0.06 | 0.04 | -0.08 | -0.06 | -0.01 | 0.01 | -0.03 |
| MB | 0.63 | 0.66 | 0.59 | 0.66 | 0.67 | 0.61 | 0.53 | 0.50 | 0.54 |
| MI | 0.18 | 0.18 | 0.23 | 0.23 | 0.22 | 0.23 | 0.23 | 0.22 | 0.16 |
| MN | -0.15 | -0.15 | 0.08 | 0.03 | 0.03 | 0.09 | 0.12 | 0.18 | 0.09 |
| MO | 0.08 | 0.06 | 0.02 | 0.10 | 0.09 | 0.03 | 0.00 | 0.09 | 0.05 |
| MT | 0.34 | 0.34 | 0.44 | 0.40 | 0.38 | 0.44 | 0.39 | 0.42 | 0.48 |
| ND | 0.27 | 0.27 | 0.33 | 0.36 | 0.36 | 0.34 | 0.35 | 0.33 | 0.27 |
| NE | 0.37 | 0.37 | 0.47 | 0.45 | 0.44 | 0.47 | 0.47 | 0.48 | 0.43 |
| NM | 0.35 | 0.23 | 0.28 | 0.39 | 0.29 | 0.28 | 0.29 | 0.30 | 0.32 |
| NY | 0.05 | 0.04 | -0.02 | 0.17 | 0.06 | -0.02 | -0.06 | -0.05 | -0.07 |
| ON | 0.19 | 0.18 | 0.26 | 0.27 | 0.26 | 0.26 | 0.27 | 0.26 | 0.20 |
| PR | 0.26 | 0.19 | 0.19 | 0.04 | -0.09 | 0.18 | 0.00 | -0.16 | -0.02 |
| SK | 0.18 | 0.21 | 0.23 | 0.06 | 0.26 | 0.23 | 0.25 | 0.26 | 0.22 |
| TX | 0.20 | 0.18 | 0.12 | 0.15 | 0.12 | 0.12 | 0.02 | 0.05 | 0.00 |
| **Mean** | **0.25** | **0.22** | **0.26** | **0.26** | **0.23** | **0.26** | **0.22** | **0.21** | **0.20** |

Supplemental Table 5: Prediction accuracy for days to flowering for each test location using BLUPs. The table includes the Pearson’s Correlation Coefficient between true and estimated values when using optimized the ensemble, bulk ensemble, and aggregate model for linear regression, ridge regression, and neural network

| Prediction Accuracy for Each Location (Days to Flowering) - BLUP-Based | | | | | | | | | |
| --- | --- | --- | --- | --- | --- | --- | --- | --- | --- |
|  | Linear Regression | | | Ridge Regression | | | Neural Network | | |
| Location | OLR | ELR | ALR | ORR | ERR | ARR | ONN | ENN | ANN |
| AB | 0.62 | 0.6 | 0.55 | 0.66 | 0.56 | 0.58 | 0.23 | 0.25 | 0 |
| CA | 0.67 | 0.62 | 0.63 | 0.64 | 0.59 | 0.63 | 0 | 0 | 0 |
| CO | 0.3 | 0.29 | -0.14 | 0.31 | 0.28 | 0.15 | -0.04 | -0.04 | 0 |
| ID | 0.03 | 0 | -0.01 | 0.02 | 0.02 | 0 | 0 | 0 | 0 |
| MB | 0.47 | 0.48 | 0.56 | 0.45 | 0.46 | 0.55 | -0.24 | -0.24 | -0.06 |
| TX | 0.26 | 0.24 | 0.35 | 0.32 | 0.28 | 0.36 | 0 | 0 | 0 |
| ND | 0.14 | 0 | -0.12 | 0.2 | 0.12 | -0.07 | 0 | 0 | 0.14 |
| NE | 0.43 | 0.42 | 0.56 | 0.38 | 0.37 | 0.53 | 0 | 0 | -0.07 |
| NY | 0.73 | 0.72 | 0.56 | 0.69 | 0.69 | 0.56 | 0.1 | 0.14 | -0.14 |
| ON | 0.7 | 0.69 | 0.3 | 0.7 | 0.7 | 0.32 | 0 | 0 | -0.43 |
| WA | 0.4 | 0.22 | 0.18 | 0.41 | 0.27 | 0.19 | 0.34 | 0.32 | 0 |
| WY | 0.38 | 0.39 | 0.09 | 0.42 | 0.38 | 0.15 | 0 | 0 | 0 |

Supplemental Table 6: Prediction accuracy for days to maturity for each test location using BLUPs. The table includes the Pearson’s Correlation Coefficient between true and estimated values when using optimized the ensemble, bulk ensemble, and aggregate model for linear regression, ridge regression, and neural network.

| Prediction Accuracy for Each Location (Days to Maturity) - BLUP-Based | | | | | | | | | |
| --- | --- | --- | --- | --- | --- | --- | --- | --- | --- |
| Location | OLR | ELR | ALR | ORR | ERR | ARR | ONN | ENN | ANN |
| AB | 0.1 | 0.1 | 0.31 | 0.26 | 0.32 | 0.29 | 0.08 | 0.27 | 0.26 |
| CA | 0.47 | 0.24 | 0.44 | 0.51 | 0.45 | 0.5 | 0.5 | 0.46 | -0.14 |
| CO | 0.12 | 0.07 | 0.14 | 0.17 | 0.14 | 0.15 | 0.1 | 0.14 | 0.14 |
| ID | 0.12 | 0.09 | 0.15 | 0.25 | 0.15 | 0.18 | 0.12 | 0.21 | 0.06 |
| KS | 0.27 | 0.21 | 0.27 | 0.18 | 0.25 | 0.16 | 0.07 | 0.21 | 0.2 |
| MB | 0.44 | 0.3 | 0.44 | 0.44 | 0.42 | 0.41 | 0.37 | 0.18 | 0.09 |
| MI | 0.39 | 0.22 | 0.29 | 0.45 | 0.3 | 0.43 | 0.2 | 0.2 | -0.16 |
| MN | 0.25 | 0.15 | 0.09 | 0.04 | 0.07 | -0.03 | 0.15 | 0.15 | 0.12 |
| MO | 0.44 | 0.29 | 0.4 | 0.3 | 0.4 | 0.29 | 0.4 | 0.3 | 0.07 |
| MT | 0.16 | 0.14 | 0.24 | 0.22 | 0.22 | 0.17 | 0.13 | 0.23 | 0.08 |
| ND | 0.37 | 0.22 | 0.42 | 0.39 | 0.42 | 0.4 | 0.25 | 0.39 | 0.2 |
| NE | 0.32 | 0.16 | 0.36 | 0.33 | 0.35 | 0.33 | 0.38 | 0.3 | 0.2 |
| NY | 0.38 | 0.13 | 0.36 | 0.45 | 0.37 | 0.4 | 0.35 | 0.34 | 0.14 |
| ON | 0.4 | 0.13 | 0.41 | 0.49 | 0.42 | 0.43 | 0.49 | 0.45 | 0.06 |
| SK | 0.13 | 0.01 | 0.02 | 0.06 | 0.01 | 0 | -0.03 | -0.08 | -0.05 |
| TX | 0.18 | 0 | 0.17 | 0.29 | 0.18 | 0.23 | 0.15 | 0.25 | 0.28 |
| WA | 0.11 | 0.11 | 0.14 | 0.22 | 0.15 | 0.13 | 0.12 | 0.21 | 0.03 |
| WY | 0.21 | 0.15 | 0.42 | 0.27 | 0.41 | 0.28 | 0.2 | 0.35 | 0.27 |

Supplemental Table 7: Prediction accuracy for seed weight for each test location using BLUPs. The table includes the Pearson’s Correlation Coefficient between true and estimated values when using optimized the ensemble, bulk ensemble, and aggregate model for linear regression, ridge regression, and neural network.

| Prediction Accuracy for Each Location (Seed Weight) - BLUP-Based | | | | | | | | | |
| --- | --- | --- | --- | --- | --- | --- | --- | --- | --- |
| Location | OLR | ELR | ALR | ORR | ERR | ARR | ONN | ENN | ANN |
| AB | 0.9 | 0.9 | 0.91 | 0.9 | 0.91 | 0.9 | 0.91 | 0.89 | 0.77 |
| AZ | 0.88 | 0.89 | 0.9 | 0.9 | 0.9 | 0.9 | 0.89 | 0.9 | 0.87 |
| CA | 0.88 | 0.88 | 0.9 | 0.9 | 0.9 | 0.89 | 0.42 | 0.15 | 0.63 |
| CO | 0.9 | 0.9 | 0.92 | 0.91 | 0.92 | 0.91 | 0.93 | 0.92 | 0.87 |
| ID | 0.88 | 0.88 | 0.91 | 0.9 | 0.91 | 0.89 | 0.9 | 0.9 | 0.8 |
| MB | 0.88 | 0.88 | 0.87 | 0.88 | 0.87 | 0.86 | 0.9 | 0.89 | 0.87 |
| MI | 0.86 | 0.86 | 0.89 | 0.89 | 0.89 | 0.89 | 0.87 | 0.8 | 0.37 |
| MN | 0.9 | 0.9 | 0.91 | 0.92 | 0.91 | 0.92 | 0.92 | 0.89 | 0.64 |
| MT | 0.85 | 0.85 | 0.88 | 0.88 | 0.88 | 0.88 | 0.9 | 0.9 | 0.87 |
| ND | 0.88 | 0.88 | 0.9 | 0.9 | 0.9 | 0.9 | 0.89 | 0.9 | 0.41 |
| NE | 0.88 | 0.89 | 0.91 | 0.91 | 0.91 | 0.91 | 0.91 | 0.89 | 0.61 |
| NM | 0.9 | 0.9 | 0.91 | 0.9 | 0.91 | 0.9 | 0.91 | 0.91 | 0.8 |
| NY | 0.9 | 0.89 | 0.91 | 0.91 | 0.91 | 0.91 | 0.9 | 0.9 | 0.76 |
| ON | 0.88 | 0.89 | 0.91 | 0.91 | 0.91 | 0.9 | 0.65 | 0.46 | 0.83 |
| SK | 0.83 | 0.88 | 0.88 | 0.87 | 0.88 | 0.87 | 0.86 | 0.87 | 0.85 |
| TX | 0.71 | 0.71 | 0.72 | 0.71 | 0.72 | 0.7 | 0.7 | 0.7 | 0.67 |
| WA | 0.88 | 0.88 | 0.91 | 0.9 | 0.9 | 0.89 | 0.83 | 0.91 | 0.83 |
| WY | 0.88 | 0.88 | 0.9 | 0.9 | 0.9 | 0.9 | 0.92 | 0.92 | 0.52 |

Supplemental Table 8: Prediction accuracy for seed yield for each test location using BLUPs. The table includes the Pearson’s Correlation Coefficient between true and estimated values when using optimized the ensemble, bulk ensemble, and aggregate model for linear regression, ridge regression, and neural network.

| Prediction Accuracy for Each Location (Seed Yield) - BLUP-Based | | | | | | | | | |
| --- | --- | --- | --- | --- | --- | --- | --- | --- | --- |
| Location | OLR | ELR | ALR | ORR | ERR | ARR | ONN | ENN | ANN |
| AB | 0.28 | 0.25 | 0.3 | 0.36 | 0.31 | 0.31 | 0.36 | 0.42 | 0.26 |
| AZ | 0.11 | 0 | 0.11 | 0.13 | 0.12 | 0.1 | 0.06 | 0.13 | 0.14 |
| CA | 0.23 | 0.2 | 0.21 | 0.27 | 0.22 | 0.21 | 0.24 | 0.16 | 0.18 |
| CO | 0.23 | 0.18 | 0.24 | 0.11 | 0.24 | 0.25 | 0.22 | 0.25 | 0.2 |
| ID | 0.34 | 0.35 | 0.31 | 0.39 | 0.32 | 0.39 | 0.26 | 0.37 | 0.38 |
| KS | 0.04 | -0.03 | -0.03 | -0.02 | -0.05 | -0.1 | -0.09 | -0.15 | -0.16 |
| MB | 0.44 | 0.46 | 0.49 | 0.49 | 0.5 | 0.49 | 0.35 | 0.34 | 0.46 |
| MI | 0.15 | 0.13 | 0.14 | 0.17 | 0.14 | 0.16 | 0.19 | 0.16 | 0.13 |
| MN | 0.01 | -0.03 | 0.14 | 0.07 | 0.14 | 0.05 | 0.03 | 0.1 | 0.02 |
| MO | 0.15 | 0.11 | 0.03 | 0.17 | 0.03 | 0.11 | 0.03 | -0.04 | -0.03 |
| MT | 0.3 | 0.27 | 0.39 | 0.35 | 0.4 | 0.35 | 0.39 | 0.38 | 0 |
| ND | 0.24 | 0.16 | 0.29 | 0.27 | 0.3 | 0.25 | 0.36 | 0.32 | 0.29 |
| NE | 0.33 | 0.32 | 0.4 | 0.39 | 0.4 | 0.38 | 0.36 | 0.4 | 0.33 |
| NM | 0.26 | 0.27 | 0.31 | 0.39 | 0.32 | 0.3 | 0.29 | 0.28 | 0.26 |
| NY | 0.07 | 0.06 | 0 | 0.17 | 0 | 0.08 | 0.07 | 0.01 | -0.04 |
| ON | 0.21 | 0.2 | 0.25 | 0.19 | 0.25 | 0.25 | 0.23 | 0.22 | 0.26 |
| PR | 0.42 | 0.25 | 0.23 | 0.4 | 0.23 | 0.18 | 0.29 | 0.28 | -0.04 |
| SK | 0.16 | 0.07 | 0.11 | 0.08 | 0.12 | 0.13 | 0.07 | 0.08 | -0.13 |
| TX | 0.27 | 0.21 | 0.14 | 0.22 | 0.14 | 0.17 | 0.01 | 0.07 | -0.11 |
| WA | 0.3 | 0.27 | 0.4 | 0.33 | 0.4 | 0.34 | 0.37 | 0.44 | 0.43 |
| WY | 0.2 | 0.2 | 0.36 | 0.31 | 0.37 | 0.31 | 0.42 | 0.39 | 0.35 |

Supplemental Table 9: For Days to Flowering: broad sense heritability (H2), genetic variance (GV), phenotypic variance (PV), residual variance (RV) and the proportion of the maximum observed variance observed across all locations present at each location (Percent Var)

| **Location** | **H2** | **GV** | **PV** | **RV** | **Percent Var** |
| --- | --- | --- | --- | --- | --- |
| AB | 0.67 | 10.40 | 19.40 | 5.10 | 0.20 |
| CO | 0.06 | 0.58 | 25.77 | 8.49 | 0.27 |
| ID | 0.08 | 16.49 | 94.95 | 25.8 | 1.00 |
| MB | 0.59 | 7.61 | 13.66 | 5.39 | 0.14 |
| ND | 0.12 | 6.38 | 77.73 | 47.74 | 0.82 |
| NE | 0.61 | 8.72 | 8.80 | 1.55 | 0.09 |
| ON | 0.34 | 9.07 | 18.56 | 0.85 | 0.20 |
| WA | 0.44 | 9.33 | 29.76 | 11.75 | 0.31 |
| WY | 0.55 | 5.00 | 7.71 | 8.47 | 0.08 |

Supplemental Table 10: For Days to Maturity: phenotypic variance (pv), borad sense heritability (H2), additive genetic variance (va), error variance (ve) and the proportion of the maximum observed across all locations present at each location (PercentVar)

|  | **H2** | **GV** | **PV** | **RV** | **Percent Var** |
| --- | --- | --- | --- | --- | --- |
| AB | 0.04 | 2.29 | 61.88 | 59.58 | 0.44 |
| CA | 0.15 | 3.42 | 22.34 | 18.92 | 0.16 |
| CO | 0.41 | 8.21 | 19.85 | 11.64 | 0.14 |
| ID | 0.18 | 4.74 | 26.28 | 21.54 | 0.19 |
| KS | 0.47 | 17.93 | 37.95 | 20.02 | 0.27 |
| MB | 0.82 | 21.86 | 26.60 | 4.74 | 0.19 |
| MI | 0.65 | 19.35 | 29.63 | 10.28 | 0.21 |
| MN | 0.03 | 4.71 | 140.77 | 136.06 | 1.00 |
| MO | 0.30 | 14.46 | 48.86 | 34.40 | 0.35 |
| MT | 0.44 | 13.22 | 30.03 | 16.81 | 0.21 |
| ND | 0.35 | 10.28 | 29.79 | 19.51 | 0.21 |
| NE | 0.50 | 18.79 | 37.46 | 18.67 | 0.27 |
| NY | 0.70 | 26.37 | 37.69 | 11.32 | 0.27 |
| ON | 0.71 | 38.53 | 54.20 | 15.67 | 0.39 |
| SK | 0.64 | 14.35 | 22.49 | 8.14 | 0.16 |
| TX | 0.23 | 6.15 | 27.04 | 20.89 | 0.19 |
| WA | 0.46 | 17.64 | 38.04 | 20.40 | 0.27 |
| WY | 0.15 | 5.87 | 39.26 | 33.39 | 0.28 |

Supplemental Table 11: For Seed Weight: phenotypic variance (pv), broad sense heritability (H2), additive genetic variance (va), error variance (ve) and the proportion of the maximum observed across all locations present at each location (PercentVar)

| **Location** | **H2** | **GV** | **PV** | **RV** | **Percent Var** |
| --- | --- | --- | --- | --- | --- |
| AB | 0.04 | 2.29 | 61.88 | 59.58 | 0.44 |
| CA | 0.15 | 3.42 | 22.34 | 18.92 | 0.16 |
| CO | 0.41 | 8.21 | 19.85 | 11.64 | 0.14 |
| ID | 0.18 | 4.74 | 26.28 | 21.54 | 0.19 |
| KS | 0.47 | 17.93 | 37.95 | 20.02 | 0.27 |
| MB | 0.82 | 21.86 | 26.60 | 4.74 | 0.19 |
| MI | 0.65 | 19.35 | 29.63 | 10.28 | 0.21 |
| MN | 0.03 | 4.71 | 140.77 | 136.06 | 1.00 |
| MO | 0.30 | 14.46 | 48.86 | 34.40 | 0.35 |
| MT | 0.44 | 13.22 | 30.03 | 16.81 | 0.21 |
| ND | 0.35 | 10.28 | 29.79 | 19.51 | 0.21 |
| NE | 0.50 | 18.79 | 37.46 | 18.67 | 0.27 |
| NY | 0.70 | 26.37 | 37.69 | 11.32 | 0.27 |
| ON | 0.71 | 38.53 | 54.20 | 15.67 | 0.39 |
| SK | 0.64 | 14.35 | 22.49 | 8.14 | 0.16 |
| TX | 0.23 | 6.15 | 27.04 | 20.89 | 0.19 |
| WA | 0.46 | 17.64 | 38.04 | 20.40 | 0.27 |
| WY | 0.15 | 5.87 | 39.26 | 33.39 | 0.28 |

Supplemental Table 12: For Seed Yield: phenotypic variance (pv), broad sense heritability (H2), additive genetic variance (va), error variance (ve) and the proportion of the maximum observed across all locations present at each location (PercentVar)

| **Location** | **H2** | **GV** | **PV** | **RV** | **Percent Var** |
| --- | --- | --- | --- | --- | --- |
| AB | 0.46 | 242,176.86 | 776,427.84 | 288,477.27 | 0.62 |
| AZ | 0.29 | 93,852.31 | 1,247,583.64 | 229,627.67 | 1.00 |
| CA | 0.18 | 72,780.19 | 726,405.65 | 328,054.38 | 0.58 |
| CO | 0.26 | 114,548.03 | 752,281.51 | 318,228.36 | 0.60 |
| ID | 0.47 | 296,137.48 | 862,075.14 | 332,006.89 | 0.69 |
| KS | 0.00 | 0.00 | 1,217,486.82 | 576,109.25 | 0.98 |
| MB | 0.57 | 242,911.30 | 430,303.15 | 184,658.71 | 0.34 |
| MI | 0.14 | 47,104.11 | 598,793.92 | 289,622.34 | 0.48 |
| MN | 0.10 | 44,573.19 | 763,080.49 | 409,715.20 | 0.61 |
| MO | 0.08 | 11,302.90 | 555,955.52 | 128,881.02 | 0.45 |
| MT | 0.44 | 139,227.77 | 717,596.50 | 180,504.93 | 0.58 |
| ND | 0.11 | 28,405.32 | 507,876.75 | 234,754.95 | 0.41 |
| NE | 0.23 | 71,637.79 | 719,724.40 | 242,977.55 | 0.58 |
| NM | 0.56 | 158,497.95 | 750,157.19 | 122,327.60 | 0.60 |
| NY | 0.26 | 49,553.95 | 409,577.12 | 143,694.83 | 0.33 |
| ON | 0.33 | 61,970.48 | 547,346.56 | 124,857.94 | 0.44 |
| PR | 0.91 | 244,482.25 | 251,555.91 | 23,125.01 | 0.20 |
| SK | 0.24 | 44,629.76 | 727,996.58 | 143,265.78 | 0.58 |
| TX | 0.09 | 6,902.06 | 449,844.54 | 66,228.19 | 0.36 |
