# Supplementary figures and images for: Environment ensemble models for genomic prediction in common bean (Phaseolus vulgaris L.)
Source: Plant Genome. 2025 Jun 12;18(2):e70057. doi: 10.1002/tpg2.70057 (PMC12159719; doi:10.1002/tpg2.70057)

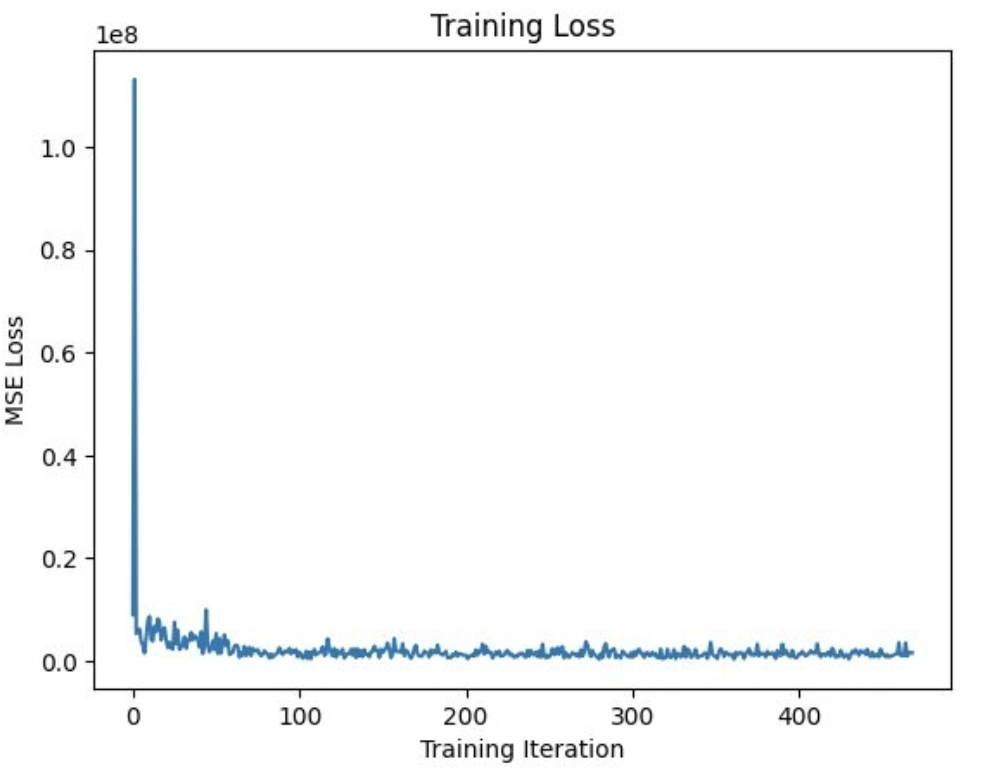

Supplement: Supplementary file 1 — Supplemental Figure 1: Training loss for a neural network. The y‐axis indicates the value for the mean squared error loss function at a given iteration, indicated on the x‐axis. The model has converged and training is complete either (1) when the loss function no longer changes beyond a given threshold, (2) or when the number of iterations is complete. In Figure 1, training was considered complete by the time 500 iterations had run. When we ran our experiments, we set the maximum number of iterations to 50 due to computational time, and the assumption below that 50 iterations is sufficient for minimizing loss. [file TPG2-18-e70057-s001.tiff]
